# Modification of dewetting characteristics for the improved morphology and optical properties of platinum nanostructures using a sacrificial indium layer

**DOI:** 10.1371/journal.pone.0209803

**Published:** 2018-12-31

**Authors:** Puran Pandey, Mao Sui, Sundar Kunwar, Sanchaya Pandit, Zenan Gu, Jihoon Lee

**Affiliations:** Department of Electronic Engineering, College of Electronics and Information, Kwangwoon University, Nowon-gu Seoul, South Korea; Institute of Materials Science, GERMANY

## Abstract

Metal nanoparticles (NPs) fabricated by means of the solid state dewetting (SSD) approach are applicable in many optoelectronic, biomedical and catalytical applications. However, the fabrication of metallic NPs with the low diffusivity elements such as platinum (Pt) has been challenging for the well-defined configuration and uniformity due to the low diffusivity of Pt atoms and thus the optical properties suffer. In this paper, the evolution of well-defined configuration and improved uniformity of Pt NPs are demonstrated by the altered solid state dewetting (ASSD) approach using a sacrificial indium (In) layer. Upon annealing, the high diffusivity In atoms can lead to the formation of In-Pt alloy due to the inter-mixing at the interface and the dewetting process advances along with the enhanced diffusion of In-Pt alloy atoms. Eventually, well-defined Pt NPs are formed by means of complete desorption of In atoms by sublimation. By the control of In and Pt ratio in the bilayers with the fixed total thickness such as In_4.5 nm_/Pt_1.5 nm_, In_3 nm_/Pt_3 nm_, In_1.5 nm_/Pt_4.5 nm_, the isolated dome shaped Pt NPs of various size are demonstrated, which reflects the significant impact of In component in the dewetting process. The optical characterization of Pt NPs exhibits the formation of quadrupolar resonance and strong dipolar resonance bands in the UV and VIS regions respectively, which are tunable based on the morphology of Pt NPs. In specific, the dipolar resonance peaks demonstrate a red shifting behavior with the increment of size of Pt NPs and gradually become narrower along with the improvement of uniformity of Pt NPs.

## Introduction

Metallic nanoparticles (NPs) have attracted extensive research interests because of their vital roles in numerous applications, i.e. optoelectronics devices [1,2], solar cells [3], fuel cells [4], sensors [5, 6], photo catalysis [7] and biomedical devices [8]. The incorporation of metallic NPs can significantly improve the optical, magnetic and catalytic properties due to the excitation of localized surface plasmon resonance (LSPR), increased surface to volume ratio, high carrier concentration and mobility, etc. [9–12]. The properties of metallic NPs can be systematically modified by the precise control of NP morphologies, namely the size, configuration, spacing and density [13, 14], which are the key components to achieve the desired performance in the related applications [15, 16]. For instance, the optical power conversion efficiency in solar cells can be significantly increased by the incorporation of Au NPs due to the increased photogenerated exciton rate by the LSPR excitation [17] and the absorption band of LSPR can be determined by the size of NPs. At the same time, the Pt NPs have been extensively applied in various catalytic reactions, hydrogen storages and optical devices due to their excellent electronic properties, chemical durability, hydrogen affinity and plasmonic resonance [18–20]. As an example, the Pt NPs embedded on anatase TiO_2_ can significantly increase the photocatalytic efficiency upon the visible light irradiation due to the efficient electron transfer between Pt NPs and TiO_2_, which promotes the O_2_ reduction and charge separation at the interface [21]. Several studies have reported the fabrication of Pt NPs [22–24] and most of them focused on improving the configuration, inter-NPs gap and uniformity without much success due to the low diffusivity as well as the high thermal stability of Pt films [25]. Generally, the fabrication of Pt NPs requires high annealing temperature (above 800°C) and the adequate surface morphology control is quite limited. Thus, the fabrication of well-defined Pt NPs with an improved uniformity and the study of morphological and optical properties can be an important foundation for the related applications. In this paper, well-defined Pt NPs with an improved uniformity is demonstrated through the altered solid state dewetting (ASSD) of bilayer system on sapphire (0001) and the corresponding LSPR properties are thoroughly explored. The introduction of In layer in between Pt and sapphire, namely the In/Pt bilayer, significantly enhances the dewetting process, which results in the formation of well-defined In-Pt NPs at relatively lower temperature as compared to the pure Pt film. The dewetting enhancement can be attributed to the high diffusivity of In atoms, inter-mixing with the Pt atoms and formation of In-Pt alloy. The sublimation of In atoms occurs throughout the dewetting process and finally, the sublimation from the alloy nanostructures matrix yields the pure Pt NPs. By the control of temperature and bilayer thickness, various size of Pt NPs can be fabricated with the improved configuration and uniformity based on the enhanced diffusion and surface energy minimization mechanism. The LSPR properties of Pt NPs are analyzed in UV–VIS–NIR regions, which exhibits the dynamic extinction, reflectance and transmittance spectra development and evolution according to the various plasmon resonance modes based on the surface morphology of Pt NPs.

## Materials and methods

In this work, the fabrication of Pt NPs was carried out on double-side polished 430 μm thick c-plane sapphire (0001) wafers with ± 0.1° off-axis (iNexus, South Korea) and the wafers were cleaved into the squares of 6 × 6 mm^2^ using a machinal saw. S1 Fig presents the morphological and optical characteristics of bare sapphire and the bare sapphire exhibited nearly flat reflectance and transmittance spectra with the ~ 13% and 85% average values respectively. Prior to the NPs fabrication, the substrates were degassed at 600°C for 30 min under 1 × 10^−4^ Torr in a pulsed laser deposition (PLD) chamber to remove trapped water vapors, particles and oxides. After the degassing, In and Pt films were deposited sequentially in a plasma-assisted sputtering chamber and the growth rate was 0.05 nm/s at the ionization current 3 mA below 1 × 10^−1^ Torr for both In and Pt films depositions. Three different series of samples were prepared with various In/Pt ratios for the fixed bilayer thickness of 6 nm such as: (i) In_1.5 nm_/Pt_4.5 nm_, (ii) In_3 nm_/Pt_3 nm_, (iii) In_4.5 nm_/Pt_1.5 nm_ as displayed in S1(E) and S1(G) Fig. For the investigation of annealing temperature (AT) effect, each sample was systematically annealed at each predefined temperature between 500 and 900°C at a ramping rate of 4°C/s under the vacuum of below 10^−4^ Torr. Previous study showed that virtually there was no diffusion below 500°C [19]. In order to maintain the consistency, the annealing process was controlled by the computer-operated recipes and at each temperature, 450 s of annealing duration was allocated to assure the adequate diffusion of adatoms. After the growth of nanostructures, the heating system was turned off and samples were kept inside within the vacuum until the temperature was reduced to an ambient. The surface morphology characterization was performed with an atomic force microscope (AFM) (XE-70, Park Systems Corp., South Korea) in a non-contact mode at an atmospheric pressure. The same batch of AFM probes was used with the specifications of a radius of curvature less than 10 nm, height 17–20 μm, force constant 40 N/m and resonant frequency ~ 270 kHz. The obtained AFM data from the XEP software was prepared in terms of top-views, color-coded side-views, cross-sectional line-profiles, histogram, Rq and SAR by the XEI software. In addition, the characterization of large-scale surface morphologies was obtained by a scanning election microscope (SEM) (CX-200, COXEM, South Korea) and the elemental analysis was performed by an energy-dispersive x-ray spectroscope (EDS) (Noran System 7, Thermo Fisher, United States of America). Finally, the optical (reflectance and transmittance) characterizations were performed with a UNIRAM II (UniNano Tech, South Korea) equipped with spectrograph (Andor Samrak sr500i) and CCD detector. The light source of halogen (450 nm ≤ λ ≤ 1100 nm) and deuterium (250 nm ≤ λ ≤ 450 nm) lamps were used to excite the samples.

## Results and discussion

**Fig 1:**
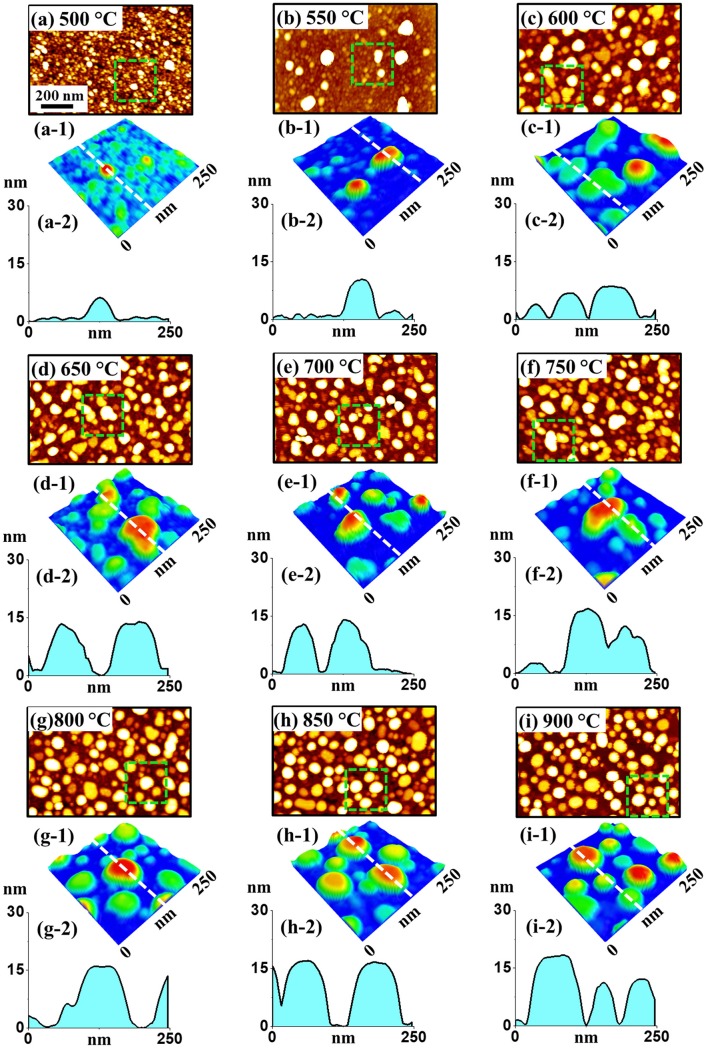
Evolution of Pt nanoparticles (NPs) from In_1.5nm_/Pt_4.5nm_ bilayer annealed between 500 and 900°C for 450 s on sapphire (0001). (a)–(i) Atomic force microscopy (AFM) top-views (1000 × 670 nm^2^). (a-1)–(i-1) Enlarged AFM color-coded side-views (250 × 250 nm^2^) of typical Pt NPs. (a-2)–(i-2) Cross-sectional line profiles of typical Pt NPs.

Fig 1 illustrates the evolution of Pt NPs fabricated with the In/Pt bilayers on c-plane sapphire though the successive annealing temperature control between 500 and 900°C for 450 s. The bilayer was consisted of 1.5 nm In and 4.5 nm Pt, denoted as In_1.5nm_/Pt_4.5nm_, with the In layer being first deposited as shown in S1(E) Fig. Generally, the evolution of Pt NPs can be divided into three regimes: i) nucleation and agglomeration of NPs, ii) irregular NPs and iii) isolated dome-shaped NPs. The formation of Pt NPs into various structural and spatial configurations can be explained based on the altered solid state dewetting (ASSD) of In/Pt bilayer along with the increased temperature. To begin with, by the conventional SSD of the pure Pt film on sapphire in the previous study [25], the formation of coalesced irregular Pt NPs with the average height and diameter of ~ 5 and 20 nm was observed with the film thickness of 5 nm. It should be pointed out that the configuration of Pt NPs was mostly coalesced and irregular, indicating that they were widely connected together without a definite shape due to insufficient diffusion even at 900°C. In contrast, the isolated spherical Pt NPs with the height and diameter of around 20 and 120 nm respectively were formed under the presence of a sacrificial In layer in this work. This sharp contrast in the NP size and configuration and further the difference in the diffusion phase can be due to the introduction of Indium atoms into the Pt matrix. Indium atoms possess much higher diffusivity and lower surface energy as compared to the Pt atoms [26] and therefore, the In atoms can diffuse even at relatively lower temperature and intermix with the Pt top-layer through the inter-diffusion. This process can result in the formation of In-Pt alloy system. As a consequence, an enhanced overall diffusivity can be induced in the system and the dewetting process can be enhanced at a much intensive phase even at comparably lower temperature. At the same time, the In adatoms can desorb from the alloy nanostructure matrix at increased temperature and can be desorbed through the sublimation, which can result in the formation of near-pure Pt nanostructures. Indium atoms can vaporize even at ~ 360°C and the vapor pressure can reach ~ 2 × 10^−6^ Torr at 600°C and ~ 5 × 10^−4^ Torr at 800°C. Along with the increased temperature, the diffusivity of alloy atoms as well as the sublimation rate can be increased, which leads to the formation of various configurations of Pt nanostructures. On top of the influence of In atoms in the diffusion kinetics, the general description of NP formation through the thermal dewetting is as follows. Upon annealing, the tiny voids or pinholes can result at the low energy sites and the voids keeps growing larger due to the surface capillary force around the rims [27]. On the other hand, the NPs start to develop due to the enhanced accumulation of atoms at elevated temperature along with the sublimation of In atoms. Finally, the fragmentation of irregular nanoclusters can take place due to the energy disorder generated by the Rayleigh instability, which results in the formation of isolated NPs [28]. At the same time, the binding energy between metallic atoms is greater than that with the substrate sapphire atoms, which leads to the 3D growth of nanostructures based on the Volmer-Weber growth model [29].

**Fig 2:**
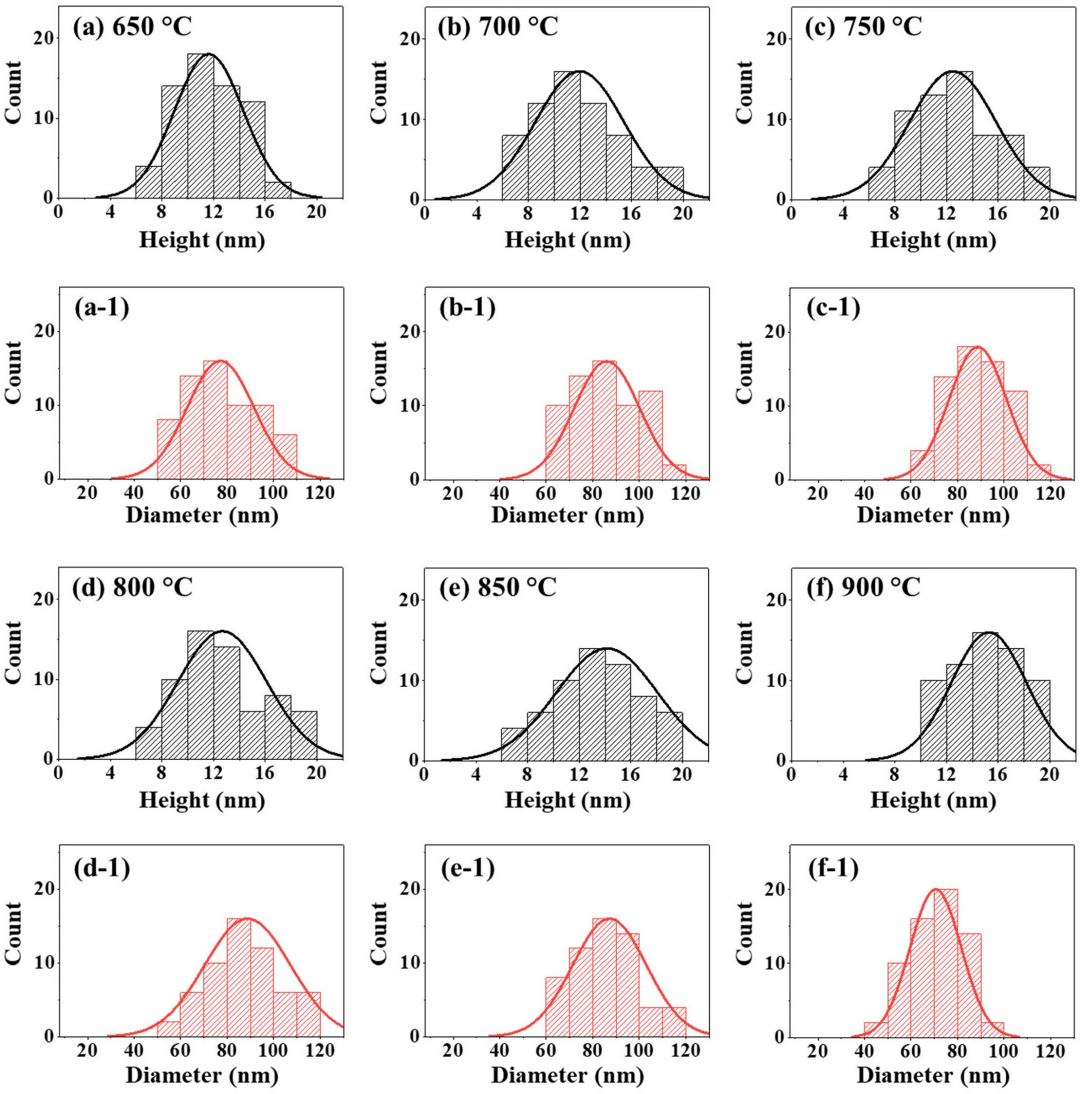
Size distribution histograms of various Pt NPs fabricated between 650 and 900 °C with In_1.5nm_/Pt_4.5nm_ bilayer. (a)–(f) Height distribution histograms. (a-1)–(f-1) Diameter distribution histogram.

**Fig 3:**
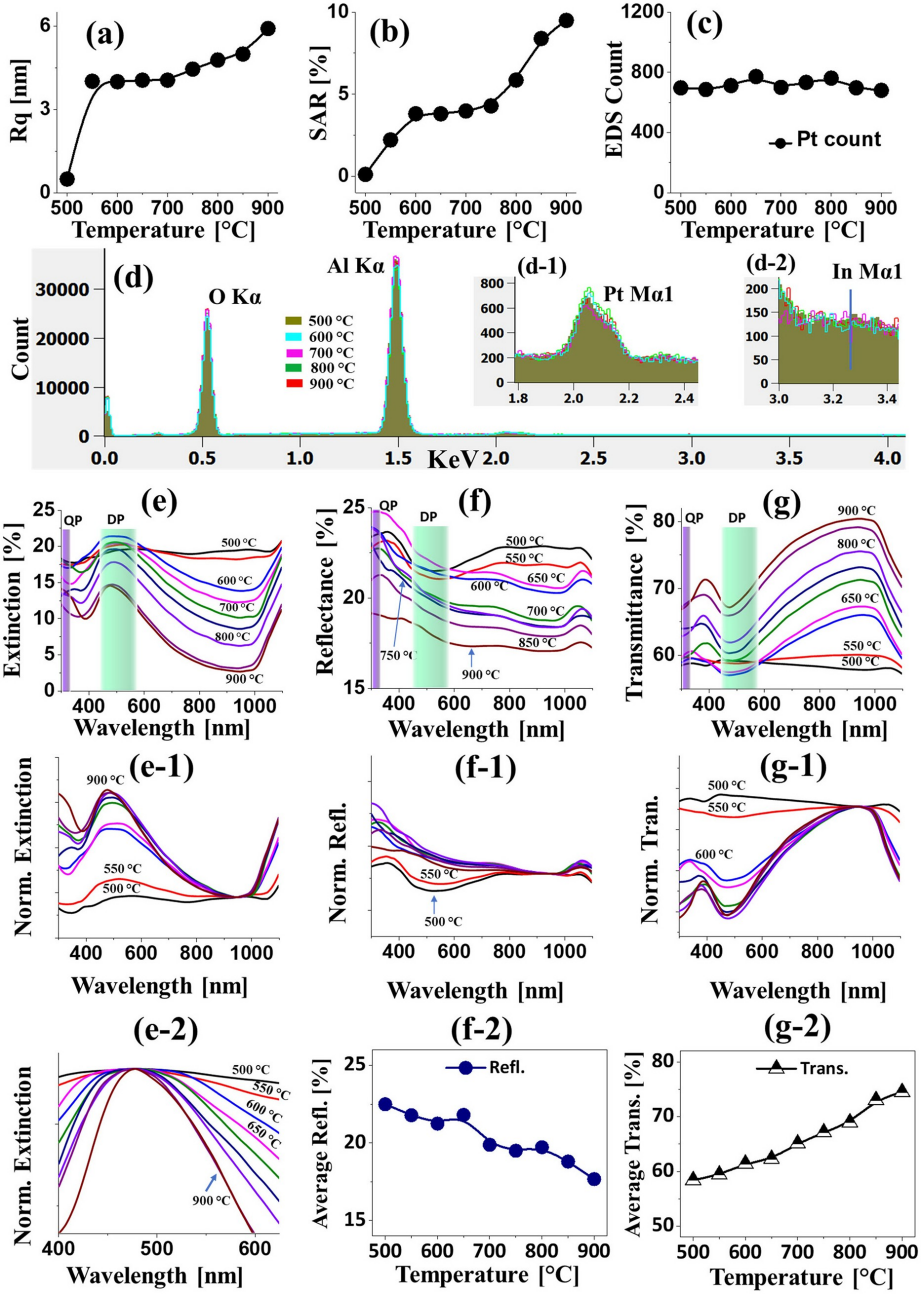
Morphological, elemental and optical analyses of Pt nanoparticles (NPs) from the In_1.5nm_/Pt_4.5nm_ bilayers. (a)–(c) RMS surface roughness (Rq), (b) Surface area ratio (SAR), (c) Energy-dispersive x-ray spectroscopy (EDS) Pt count of various Pt nanostructures from the In_1.5nm_/Pt_4.5nm_ bilayer. (d) EDS spectra. (d-1)—(d-2) Enlarged Pt and In spectra. (e)–(e-2) Extinction, normalized extinction and enlarged extinction spectra. (f)–(f-2) Reflectance, normalized reflectance and plot of average reflectance. (g)–(g-1) Transmittance, normalized transmittance and plot of average transmittance.

The detailed surface morphology characterization of Pt NPs is presented with the AFM top-views in Fig 1(A)–1(I), enlarged color-coded AFM side-views in Fig 1(A-1)–1(I-1), line profiles in Fig 1(A-2)–1(I-2) and corresponding size distribution histograms in Fig 2. In specific, tiny Pt nanostructures were formed at low energy sites due to the diffusion of In-Pt atoms and the concurrent sublimation of In atoms at 500°C as shown in Fig 1(A)–1(A-1). The size of typical tiny structures is ~ 4 nm (height) and ~ 30 nm (diameter) as shown in Fig 1(A-2). At 550°C as shown in Fig 1(B), few irregular Pt hillocks of height and diameter ~ 12 and 45 nm respectively were formed, which can be due to the enhanced diffusion of In-Pt atoms with the increased temperature. With the further increase in temperature to 600°C, the Pt hillocks were drastically transformed into the isolated-irregular Pt NPs where the dimension was also significantly enlarged such that the average height and diameter were ~ 10 nm and ~ 60 nm respectively as shown in Fig 1(C)–1(C-1). From temperatures 650°C to 800°C, the irregular Pt NPs were gradually transformed into the semi-spherical configuration along with the size increment to ~ (14–18) nm in height and ~ (80–100) nm in diameter as shown in Fig 1(D)–1(G) and 3D side-views Fig 1(D-1)–1(G-1). Finally, at high temperature of 850 and 900°C, the Pt NPs of mostly semi-spherical shape of ~ 16 nm height and ~ 100 nm diameter were obtained as observed in Fig 1(H) and 1(I). The semi-spherical shape and uniform size of Pt NPs can be developed due to the isotropic distribution of surface energy as well as surface energy minimization [30]. Furthermore, the size and shape uniformity were largely improved and the gap between NPs was further increased. In this case, the Pt NPs generally possessed the semi-spherical/dome shape, which can be correlated to the equilibrium configuration of NPs to become thermodynamically stable [31]. These results clearly demonstrated a significant improvement in the size, shape and uniformity of Pt NPs in comparison with the previous studies, which can be attributed to the diffusion enhancement by the In component.

Furthermore, the morphological analysis of Pt NPs between 650 and 900°C are studied in terms of height and diameter distribution histogram as shown in Fig 2. In general, the height distribution histogram showed a relatively narrower distribution at 650°C and it was wider with the increased annealing temperature as shown in Fig 2(A)–2(F). As a consequence, the average height of Pt NPs was gradually increased from 11.6 to 15.3 nm with the increased annealing temperature from 650 to 900°C. Meanwhile, the diameter distribution histogram became wider between 650 to 850 °C with the formation of elongated Pt NPs and it was narrower with the formation of round Pt NPs at 900°C. Consequently, the average diameter of Pt NPs was gradually increased from 77.1 to 87.2 nm between 650 and 850°C and slightly reduced to 70.5 nm at 900°C. In addition, the morphology evolution of Pt NPs can also be witnessed by the RMS roughness (Rq) and surface area ratio (SAR) plots as shown in Fig 3(A)–3(B) and S1 Table. The Rq was 0.5 nm at 500°C and it was sharply increased to 3.21 nm at 550°C due to the transformation of the Pt tiny structures to large Pt hillocks. Then, it mildly increased (3.99–5.91 nm) between 600 and 900°C along with the increment of the vertical size of isolated Pt NPs as described above. Similarly, the SAR demonstrated a similar trend along with the evolution of Pt NPs at specific temperature. The SAR value was 0.12% at 500°C which was increased sharply to 3.81% at 600°C. Afterward, the SAR value was gradually increased up to 9.49% between 650°C and 900°C with the growth of isolated and regular Pt NPs. The EDS analysis of Pt NPs at different temperature are presented in Fig 3(C) and 3(D) with the EDS summary count and spectra. From the EDS spectra, the high intensity Kα peaks of substrate elements O and Al were observed at 0.53 and 1.49 KeV respectively. The Pt Mα1 was detected at 2.049 KeV for all samples between 500 and 900°C with similar peak counts as displayed in Fig 3(D-1), which indicates the constant amount of Pt throughout the temperature range. The EDS count of In at 3.3 KeV was not detected as shown in Fig 3(D-2) due to the extensive sublimation. This indicates that the as fabricated NPs were mostly pure Pt in which the In atoms assisted the dewetting process and sublimated at the end.

The LSPR properties of corresponding Pt NPs in terms of extinction, reflectance and transmittance spectra are presented in Fig 3(E)–3(G). In which, the reflectance and transmittance spectra were experimentally measured at normal incidence of light and the extinction spectra were extracted by following relation: reflectance [%] + transmittance [%] + extinction [%] = 100 [%]. Depending upon the size, configuration, uniformity and surface coverage of Pt NPs at increased temperature, the optical behaviors were also correspondingly evolved. In particular, the extinction spectra in Fig 3(E) clearly exhibited two absorption peaks: one weak peak at the UV region and the other intense and broad peak at VIS region, which can be correlated to the excitation of various LSPR band of Pt NPs [32]. Generally, the fabricated Pt NPs are below 100 nm in size with semi-spherical configuration, and thus the UV and VIS absorption peaks can be induced by the quadrupolar (QP) and dipolar (DP) resonance modes of Pt NPs respectively [32]. With the small Pt NPs, the DP resonance was found to be more pronounced, which results in the stronger absorption at the VIS region as compared to the UV region. In addition, the extinction spectra were normalized at 950 nm to elucidate the NPs size behavior as shown in Fig 3(E-1). With the increased size of Pt NPs at high temperature, the intensity of absorption peaks was gradually increased, signifying the enhanced absorption with larger Pt NPs. At the same time, the DP resonance peaks demonstrated a gradual narrowing effect for high temperature samples as shown in Fig 3(E-2), which can be correlated the improved NPs uniformity due to the enhanced diffusion at increased temperature [33]. The corresponding reflectance spectra of Pt NPs, normalized reflectance spectra and plot of average reflectance are shown in Fig 3(F)–3(F-2). In particular, for the compact and wide coverage Pt NPs below 550°C, the reflectance spectra demonstrated the broad absorption dip at VIS region (~ 520 nm) whereas for the comparatively larger and isolated Pt NPs at higher annealing temperature, the shoulder was formed in the same region. As discussed, for the small size Pt NPs the DP resonance mostly contributed the absorption in the VIS region. However, with the definite formation of Pt NPs, the backscattering effect can also be enhanced, which can result in the overlapping of absorption dip with the development of shoulder at VIS region as shown in Fig 3(F-1) [34]. The reflectance was gradually reduced with the increased temperature likely due to the reduced surface coverage of Pt NPs, which is clearly demonstrated by the plot in Fig 3(F-2). Furthermore, the transmittance spectra in Fig 3(G)–3(G-1) generally demonstrated the dipolar and quadrupolar absorption bands in the VIS and UV region respectively. However, in the case of high coverage tiny features between 500 and 550°C, the absorption dips were not obvious, which can be affected by the forward scattering of Pt NPs. When the isolated and larger Pt NPs were formed, the absorption dips were gradually enhanced as shown in Fig 3(G-1). In terms of average transmittance, it showed the opposite behavior with the reflectance: i.e. the transmittance was increased along with the reduced surface coverage of Pt NPs. From the optical analysis of Pt NPs in this set, it was found that the tunable optical properties can be achieved with the proper control of Pt NPs. Furthermore, the VIS absorption bands and its tunability was significantly improved from the previous studies with non-uniform and random Pt NPs.

**Fig 4:**
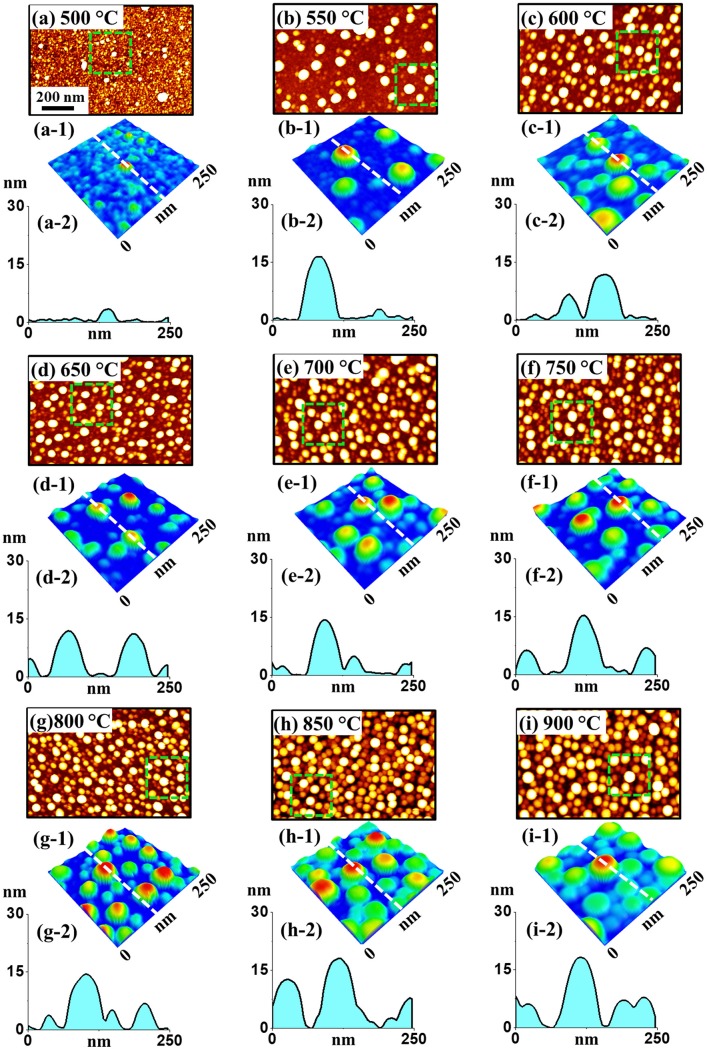
**Relatively smaller Pt NPs with In**_**3 nm**_**/Pt**_**3 nm**_ **bilayers by annealing between 500 to 900°C for 450 s on sapphire (0001).** (a)–(i) AFM top-views (1000 × 670 nm^2^). (a-1)–(i-1) Enlarged color-coded side-views (250 × 250 nm^2^). (a-2)—(i-2) Cross-sectional line profiles acquired from the lines in (a-1)—(i-1).

**Fig 5:**
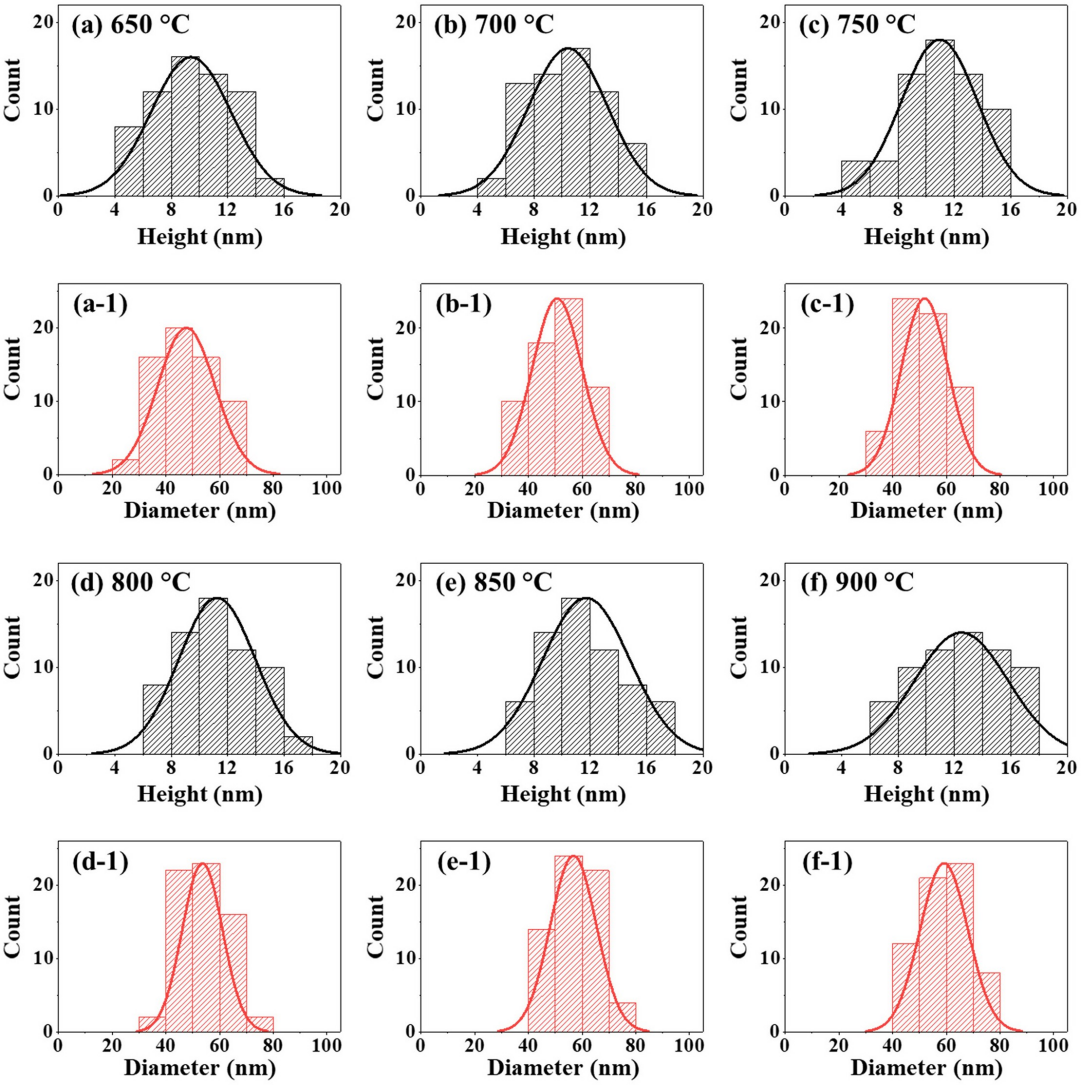
**Size distribution histograms analysis of various Pt NPs fabricated with the with the In**_**3 nm**_**/Pt**_**3 nm**_ **bilayers.** (a)–(f) Height distribution histogram of various Pt NPs 650 and 900 °C. (a-1)–(f-1) Corresponding diameter distribution histogram of Pt NPs.

**Fig 6:**
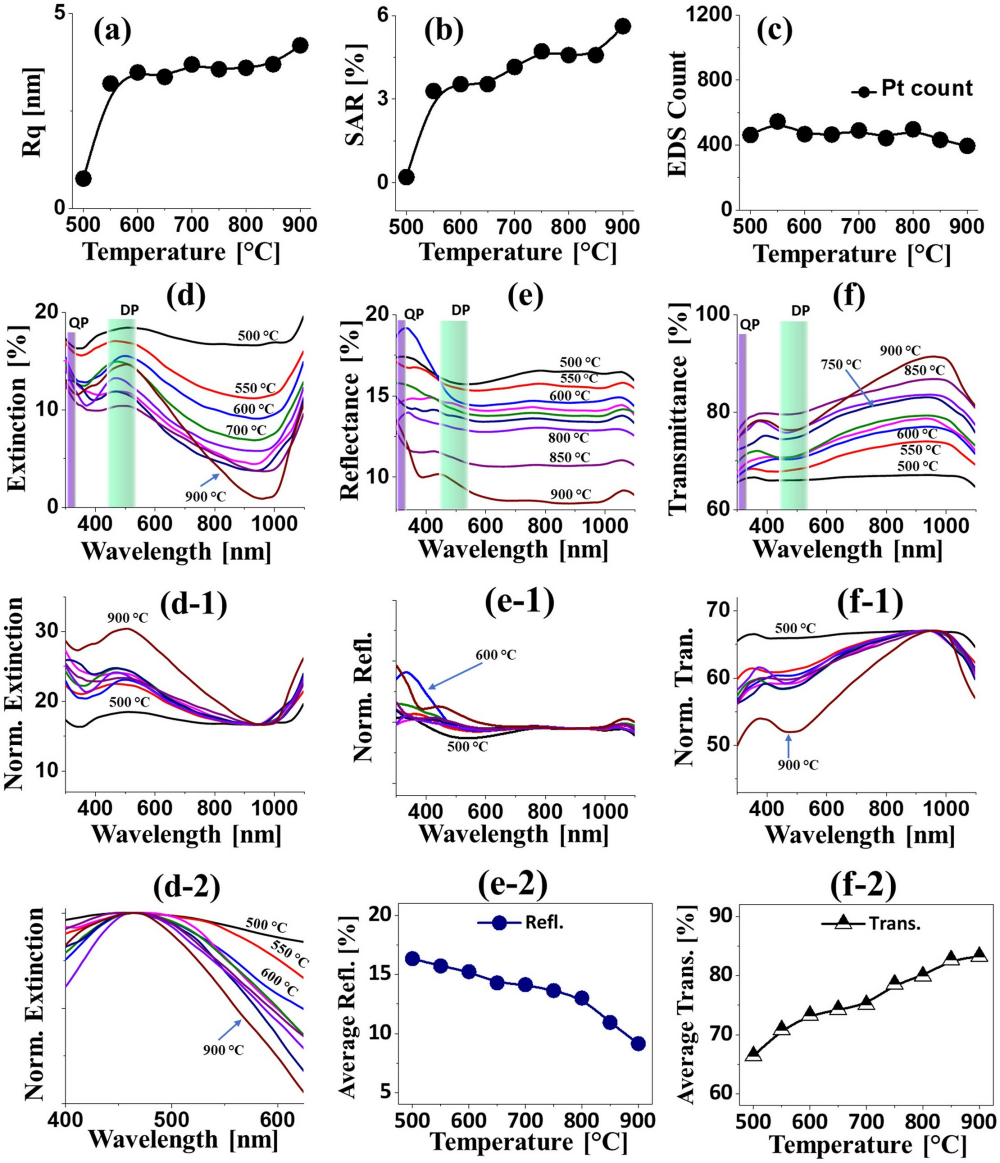
Morphological, elemental and optical analyses of the In_3 nm_/Pt_3 nm_ series. (a)–(c) Summary plots of Rq, SAR and EDS count of Pt nanostructures with the In_3 nm_/Pt_3 nm_ bilayers as function of temperature. (d)–(d-2) Extinction, normalized extinction and magnified extinction spectra. (e)–(e-2) Reflectance, normalized reflectance and average reflectance. (f)–(f-2) Transmittance, normalized transmittance and average transmittance.

Fig 4 shows the fabrication of Pt NPs with the distinct bilayer thickness of In_3 nm_/Pt_3 nm_ at an identical growth condition with the previous set (annealing between 500 and 900°C for 450 s). The AFM analysis in terms of top-views, color-coded side-views and cross-sectional line-profiles are demonstrated in Fig 4 and that of height and diameter distribution histogram are presented in Fig 5. As compared to the previous set, the indium thickness was increased whereas the Pt thickness was decreased while keeping the total thickness of bilayer the same at 6 nm. With this individual layer thickness variation, the dewetting process was significantly altered, which resulted in the formation of more regular and denser Pt NPs with the reduced size as seen Fig 4(A)–4(I). The dewetting process was significantly enhanced because of the increased amount of In, which possess a higher diffusivity [26]. At the same time, by reducing the Pt layer, the dewetting can be further enhanced as the stability of Pt film against dewetting can be minimized [35]. Meanwhile, the inter-mixing as well as alloying between In and Pt atoms can be increased at the interface because of the high amount of In atoms, which can enhance the overall diffusivity of the system. Eventually, numerous voids as well as alloy NPs can be nucleated at low energy sites at relatively lower temperature due to the higher diffusivity of In-Pt atoms. Along with the increased temperature, In atoms can gradually sublimate from the alloy NPs and therefore nearly pure Pt NPs was obtained as discussed. In specific at 500°C, the continuous In/Pt bilayer transformed to a rougher surface having small pinholes and tiny Pt structures due to the limited diffusion of In-Pt atoms as shown in AFM top-views in Fig 4(A) and color-coded side-view 4(a-1). Larger isolated NPs were drastically evolved when the temperature was increased from 500 to 650°C due to the enhanced diffusion of alloy atoms as shown in Fig 4(D)–4(D-2). In specific, the height and diameter distribution histogram presented in Fig 5(A)–5(A-1) demonstrated the height of Pt NPs was dispersed between 4–16 nm and that of diameter was widely distributed in the range of 20–70 nm. Consequently, the average height and diameter of the Pt NPs were 9.4 and 47.6 nm respectively. Subsequently, between 700 to 900°C, the isolated round Pt NPs with increased size and improved uniformity were evolved along with the enhanced diffusion as shown in Fig 4(E)–4(I). Within this temperature range, the average height and diameter of Pt NPs were slightly increased as shown in Fig 5(B)–5(F) and 5(B-1)– 5(F-1). In particular, at 900°C, the height and diameter distribution of Pt NPs were slightly higher ranger such that height range was 6–8 nm and diameter range was 40–80 nm. This resulted the increased average height and diameter of Pt NPs to 12.4 and 59.1 nm respectively. The overall evolution of Pt NPs can be related to the surface energy minimization mechanism to attain the equilibrium configuration as discussed [31]. Under this growth condition, the Pt NPs showed similar vertical height while the diameter was slightly reduced as compared to the previous set. Furthermore, the areal density of Pt NPs in this set is highly increased, which can be due to the enhanced dewetting of In/Pt bilayer with the increased amount of In. Moreover, the morphological transformation was studied in terms of Rq and SAR as displayed in Fig 6(A) and 6(B) and S1 Table. The Rq was ~ 0.76 nm at 500°C, which abruptly increased by ~ 4 times to 3.2 nm at 550°C due to the formation of larger Pt NPs. Later, there was a mild increment in the Rq to 4.18 nm till 900°C as the height of NPs was slightly increased. A very similar trend was observed in the case of SAR with respect to temperature. The SAR value was 0.2% at 500°C, which abruptly increased to 3.29% at 550°C and gradually increased to 5.61% at 900°C along with the gradual higher surface area exerted by the Pt NPs. On the other hand, the EDS count plot in Fig 6(C) indicated the presence of similar amount of Pt at various annealing temperature and the In peak was not detected throughout the range due to the sublimation.

The LSPR characteristics of the relatively smaller Pt NPs is presented in Fig 6(D)–6(F) along with their corresponding normalized spectra. Generally, the optical data showed similar spectral trend with previous set, however, due to the variation in size, configuration and uniformity, the absorption intensity, position and bandwidth were significantly varied from the previous set. In the case of extinction spectra as shown in Fig 6(D), two peaks in the UV and VIS regions corresponding to the QP and DP resonance effect of small Pt NPs were commonly observed. As compared to previous set In_1.5nm_/Pt_4.5nm_ (from ~ 560 nm to ~ 480 nm), the extinction peaks were generally at shorter wavelength (from ~ 510 to ~ 470 nm) and average absorption was lower due to the formation of smaller Pt NPs [33]. As the small Pt NPs became comparatively larger with temperature, the absorption was slightly increased as shown by the normalized spectra (normalized at 950 nm) in Fig 6(D-1). At the same time, the DP resonance peak was gradually narrowed with increased temperature as shown in the magnified spectra in Fig 6(E-2) due the improvement of size uniformity of Pt NPs [33]. The reflectance spectra of small Pt NPs in Fig 6(E)–6(E-1) generally demonstrated a shoulder at the UV-VIS region and flat spectral shape at longer wavelength. Generally, the absorption dips at UV and VIS region can be expected corresponding to the QP and DP resonance, but due to the enhanced backscattering with the DP of the small Pt NPs, the absorption dip can be reduced or distorted [34]. Meanwhile, the average reflectance was decreased as a function of annealing temperature due to the reduced surface coverage of Pt NPs as shown by the original reflectance spectra and plot in Fig 6(F) and 6(F-2). In the transmittance spectra as shown by the Fig 4(F), two absorption dips in the UV and VIS region associated with QP and DP were clearly observed. And, the absorption dips were gradually enhanced with the formation of relatively larger Pt NPs at higher temperature as shown by the Fig 6(F-1). In terms of average transmittance, it was increased due to the reduced surface coverage with temperature as presented in Fig 6(F-2).

**Fig 7:**
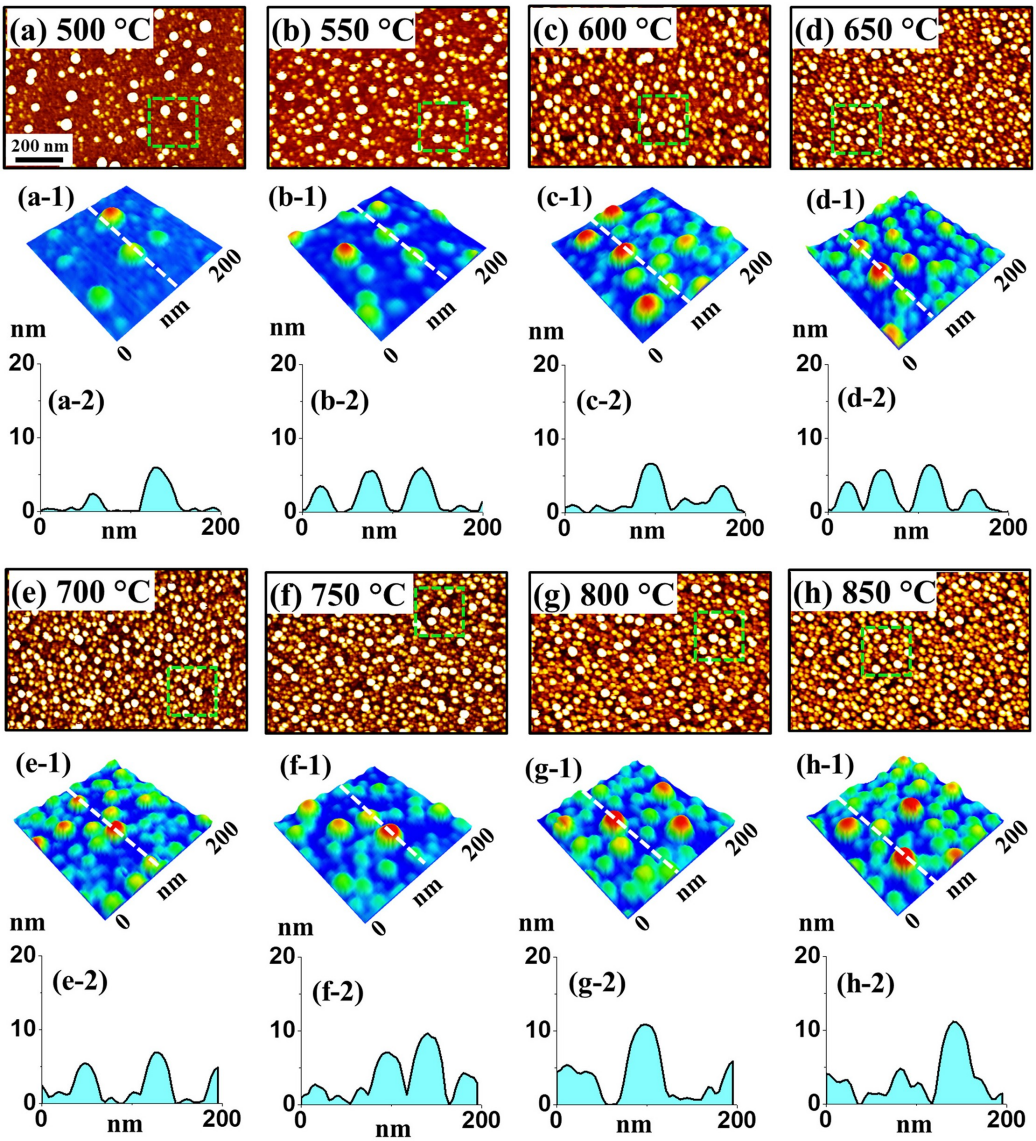
Densely packed small Pt NPs fabricated with In_4.5 nm_/Pt_1.5 nm_ bilayers by the annealing between 500 and 850°C for 450 s on sapphire (0001). (a)–(h) AFM top-views (1000 × 670 nm^2^). (a-1)–(h-1) Enlarged color-coded side-views (200 × 200 nm^2^). (a-2)–(h-2) Corresponding cross-sectional line profiles.

**Fig 8:**
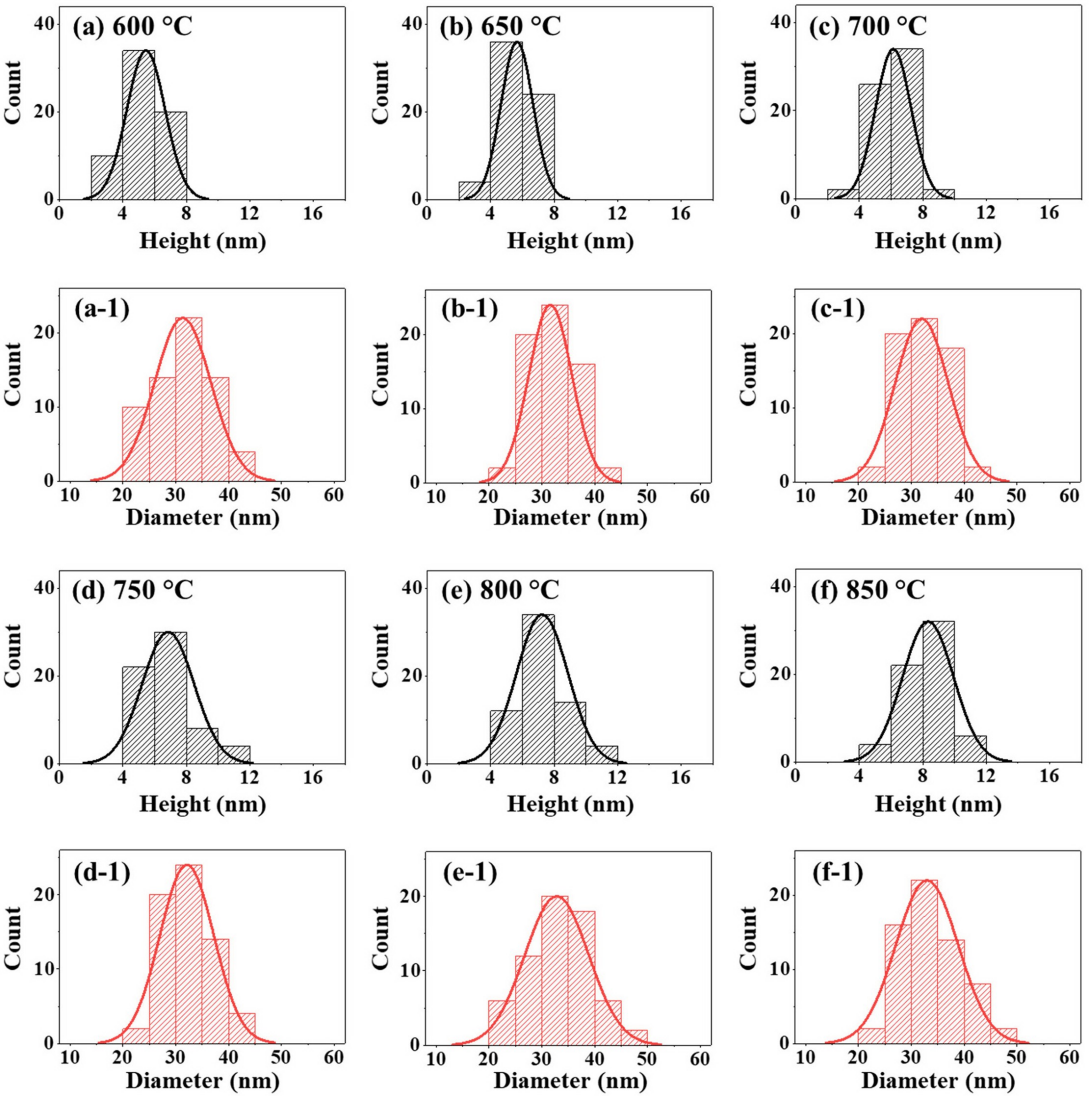
Morphological analysis of Pt NPs fabricated with the In_4.5 nm_/Pt_1.5 nm_ bilayers in terms of height and diameter distribution histograms. (a)–(f) Height distribution histogram and (a-1)–(f-1) diameter distribution histogram of various Pt NPs at temperature between 600 and 850°C.

**Fig 9:**
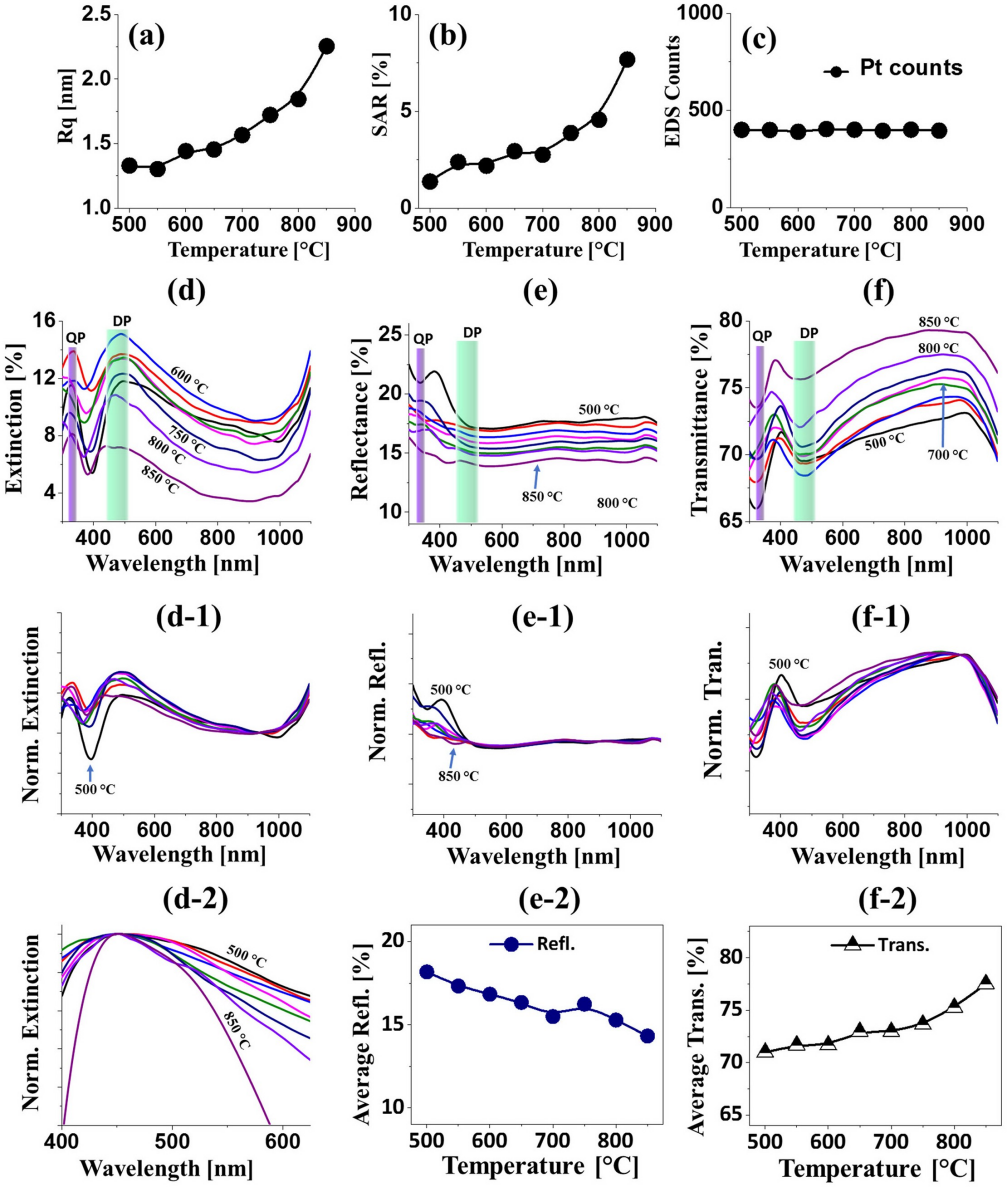
Morphological, elemental and optical analyses of the In_4.5 nm_/Pt_1.5 nm_ set. (a)–(c) Summary plots of Rq, SAR and EDS count of small Pt NPs fabricated with the In_4.5nm_/Pt_1.5nm_ bilayers. (d)–(d-2) Extinction, normalized extinction and magnified extinction spectra. (e)–(e-2) Reflectance, normalized reflectance spectra and plot of average reflectance. (f)–(f-2) Transmittance, normalized transmittance spectra and plot of average transmittance.

Fig 7 shows the evolution of Pt NPs with the In_4.5 nm_/Pt_1.5 nm_ bilayer series. The total thickness (6 nm) of In/Pt bilayer and the growth conditions (between 500 and 900°C for 450 s) were same as the previous two sets. However, the thickness of In and Pt were 4.5 nm and 1.5 nm respectively, which is just reversed as compared to the first set In_1.5 nm_/Pt_4.5 nm_. The morphological evolution of Pt NPs at this condition exhibited clear differences due to the variation of individual deposition thickness of In and Pt bilayer as can be expected. In this case, the stability of Pt layer against dewetting was further decreased due to the reduced Pt thickness as discussed [36]. Due to the higher amount of In layer, the significant intermixing of In atoms with Pt atoms can occur and this can result in the enhanced overall dewetting of In/Pt bilayer and growth of the Pt NPs along with the sublimation of In atoms as discussed. As a result, the degree of dewetting extent can be shifted, which could produce definite NPs even at lower annealing temperature. In specific, at 500°C highly compact tiny Pt nanostructures were formed along with the few larger NPs as shown in Fig 7(A) due to the comparatively enhanced diffusivity of atoms with increased percentage of In component. The surface morphology was drastically changed at 550 and 600°C with the formation of isolated Pt NPs as shown in Fig 5(B) and 5(C), which can be attributed to the enhanced diffusion as well as sublimation of In atoms as discussed. When the temperature was increased between 650 and 850°C, the evolution of isolated NPs was more pronounced as observed in Fig 5(C)–5(H). The height and diameter distribution of Pt NPs presented in Fig 8 showed a slight variation, which can be due to the formation of thermally stabilized NPs of critical size and structure with the given thickness [36]. In specific, the height distribution was slightly varied from 2–8 nm to 4–12 nm when the annealing temperature was increased from 600 to 900°C. Similarly, the mild diameter distribution was observed such that 20–45 nm at 600°C and slightly increased to 20–50 nm at 900°C. As a consequence, the average height and diameter of Pt NPs fabricated at 600°C were 5.4 and 31.3 nm respectively. The average height and diameter was slightly increased to 8.3 and 32.9 nm respectively at 900°C along with the formation of comparatively larger Pt NPs. By comparing with the first set In_1.5 nm_/Pt_4.5 nm_, the average height and diameter of Pt NPs were reduced by nearly two times whereas the number density was further increased. The Rq and SAR were consistently elevated from around 1.3 to 2.2 nm and 1.3 to 7.6% respectively when the temperature was increased from 500 to 850°C as shown in Fig 9(A) and 9(B) and S2 Table. And the corresponding elemental analysis in Fig 9(C) indicated the constant Pt peak and the NPs are of only Pt. The optical characterization of very small Pt NPs is shown in Fig 9(D)–9(F) along with the extinction, reflectance and transmittance. Generally, the Pt NPs in this set were much smaller and denser as compared to the previous sets, which resulted in slightly variant optical properties. As in the previous sets, the extinction spectra depicted two absorption peaks at UV and VIS region, which corresponds to the QP and DP resonance modes as shown in Fig 9(D). The DP resonance peak showed a slight blue shift as compared to the previous result [35] and further the absorption intensity was further reduced due to the much smaller size of Pt NPs. From the normalized extinction spectra (normalized at 950 nm) in Fig 9(D-1), the absorption intensity was slightly increased as the evolution of NPs was minor with temperature. Meanwhile, the absorption band width was gradually narrowed as in the previous cases due to the improved size uniformity as discussed [33]. In terms of reflectance spectra, it demonstrated a shoulder in the UV-VIS region and a flat spectral shape at longer wavelength as shown in Fig 9(E) and 9(E-1). The formation of shoulder at UV-VIS region instead of the absorption dip can be correlated to the enhanced backscattering with the strong DP resonance of small NPs [34]. The transmittance spectra in Fig 9(F) and 9(F-1) showed the formation of UV and VIS dips for all samples and the absorption dips were also slightly blue shifted as compared to the previous cases. As the size of Pt NPs were mildly increased with temperature, the transmittance dips were also intensified indicating stronger absorption. In terms of average reflectance and transmittance, they showed a similar trend as shown by the plots in Fig 9(E-2)–9(F-2).

## Conclusion

In summary, the fabrication of Pt NPs of various size, density and spacing on c-plane sapphire (0001) have been successfully demonstrated by the altered solid state dewetting (ASSD) of the Pt films using a sacrificial In layer. The dewetting of Pt NPs was significantly improved by adding the In layer having low surface energy, high diffusivity and sublimation rate. Upon annealing, the inter-mixing between In and Pt atoms at the In/Pt interface formed the In-Pt alloy, which subsequently enhanced the overall diffusion and thus the dewetting process. The formation of well-developed Pt NPs was realized along with the sublimation of In atoms and dewetting at increased temperature. The Pt NPs fabricated in this work showed improved uniformity, shape and size, which were obtained at relatively lower temperatures as compared to the conventional dewetting of Pt on sapphire. By varying the individual thickness of In and Pt at a constant total thickness of 6 nm such as In_1.5 nm_/Pt_4.5 nm_, In_3 nm_/Pt_3 nm_ and In_4.5 nm_/Pt1_.5 nm,_ various size and configuration of Pt NPs were obtained, which inevitably provided the additional opportunity to tune the surface morphology of Pt NPs. In addition, the Pt NPs demonstrated the strong LSPR band in the UV and VIS wavelength corresponding to the excitation of different LSPR modes. In specific, a large LSPR enhancement in the visible wavelength was observed due to the strong dipolar resonance whereas a relatively weaker LSPR band was obtained in the UV region due to the quadrupolar resonance mode. The dipolar resonance band was found to be more dynamic and sensitive to the surface morphology change of the Pt NPs.

## Supporting information

S1 Fig (a) AFM surface morphology of bare sapphire (0001). (b) Cross-sectional line profile from the lines in (a). (c)–(d) Transmittance (T) and reflectance (R) spectra of bare sapphire. (e)–(g) Schematic images of various In/Pt bilayer deposition as labelled.
(DOCX)

S2 Fig AFM top-views (3 × 3 μm^2^) of samples after the deposition of In-Pt bilayers with various composition. (a) In_1.5 nm_/Pt_4.5 nm_, (b) In_3 nm_/Pt_3 nm_ and (c) In_4.5 nm_/Pt_1.5 nm_. (a-1)–(c-1) Corresponding cross-sectional profiles.
(DOCX)

S3 Fig Effect of annealing temperature (500°C– 650°C) on the morphological evolution of Pt NPs on sapphire (0001) with the In_1.5 nm_/Pt_4.5 nm_ bilayer. (a)–(d) AFM 3D side-views (1 × 1 μm^2^). (a-1)–(d-1) Cross-sectional line profiles.
(DOCX)

S4 Fig Morphological evolution of Pt NPs on sapphire (0001) with the In_1.5 nm_/Pt_4.5 nm_ bilayer at various annealing temperature (700°C– 900°C). (a)–(e) AFM 3D side-views (1 × 1 μm^2^). (a-1)–(e-1) Cross-sectional line profiles.
(DOCX)

S5 Fig Full range energy-dispersive x-ray spectroscope (EDS) spectra of Pt NPs on sapphire with the In_1.5 nm_/Pt_4.5 nm_ bilayer annealed between 500 and 900°C for 450 s. The enlarged views of Pt Mα1 is presented as insets.
(DOCX)

S6 Fig Evolution of self-assembled Pt NPs on sapphire (0001) with the fixed bilayer total thickness of 6 nm (In_3 nm_/Pt_3 nm_) and annealing between 500 and 650 oC for 450 s. (a)–(d) AFM D side-views (1 × 1 μm^2^). (a-1)–(d-1) Cross-sectional line profiles.
(DOCX)

S7 Fig Self-assembled Pt NPs on sapphire (0001) with the fixed bilayer total thickness of 6 nm (In_3 nm_/Pt_3 nm_) by the annealing between 700 and 900 oC for 450 s. (a)–(e) AFM D side-views (1 × 1 μm^2^). (a-1)–(e-1) Cross-sectional line profiles.
(DOCX)

S8 Fig EDS spectra of the Pt NPs on sapphire within the range of 0–5 keV annealed at temperatures from 500 to 900°C for 450 s with the In_3 nm_/Pt_3 nm_ bilayer set. Insets show the detail of the Pt Mα1 peak.
(DOCX)

S9 Fig Uniform Pt NPs on sapphire (0001) by the systematic control of annealing temperature from 700 to 850°C for 450 s. The total bilayer thickness was of 6 nm with the In_4.5 nm_/Pt_1.5 nm_ bilayer. (a)–(d) AFM side-views (1 × 1 μm^2^). (a-1)–(d-1) Cross-sectional line profiles.
(DOCX)

S10 Fig EDS spectra of the Pt NPs on sapphire, fabricated with various annealing temperature as labeled with the In_4.5 nm_/Pt_1.5 nm_ bilayer. The insets show the enlarged Pt Mα1 peaks at each temperature.
(DOCX)

S1 Table Summary of root mean squared roughness (Rq), surface area ratio (SAR), average reflectance and transmittance of Pt NPs fabricated between 500 and 900°C for 450 s with 6 nm total thickness (In_1.5 nm_/Pt_4.5 nm_). (DOCX)

S2 Table Summary of Rq, SAR, average reflectance and transmittance of Pt NPs fabricated at temperature 500–900°C for 450 s with the In_3 nm_/Pt_3 nm_ bilayer on sapphire (0001). (DOCX)

S3 Table Summary of Rq, SAR, average reflectance and transmittance of Pt nanostructures fabricated between temperature 500–850°C for 450 s with the In_4.5 nm_/Pt_1.5 nm_ bilayer on sapphire (0001). (DOCX)
