# Supplementary material for: Modification of dewetting characteristics for the improved morphology and optical properties of platinum nanostructures using a sacrificial indium layer
Source: PLoS One. 2018 Dec 31;13(12):e0209803. doi: 10.1371/journal.pone.0209803 (PMC6312214; doi:10.1371/journal.pone.0209803)
Supplement: S1 Fig — (a) AFM surface morphology of bare sapphire (0001). (b) Cross-sectional line profile from the lines in (a). (c)–(d) Transmittance (T) and reflectance (R) spectra of bare sapphire. (e)–(g) Schematic images of various In/Pt bilayer deposition as labelled. (DOCX) [file pone.0209803.s001.docx]

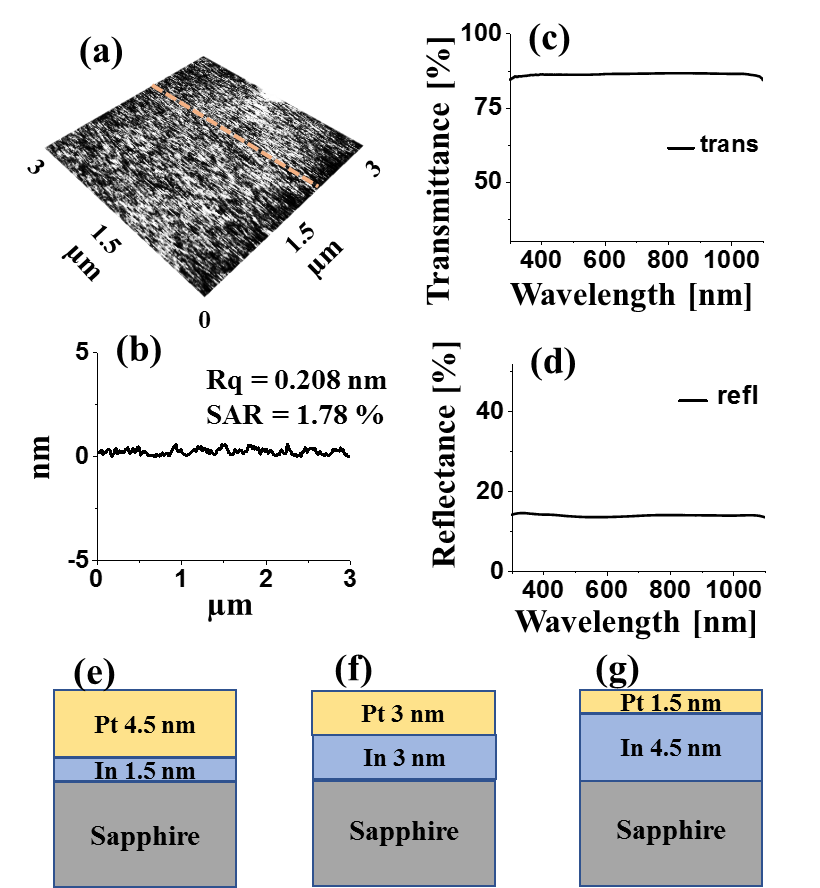


**S1 Fig.** (a) AFM surface morphology of bare sapphire (0001). (b) Cross-sectional line profile from the lines in (a). (c) – (d) Transmittance (T) and reflectance (R) spectra of bare sapphire. (e) – (g) Schematic images of various In/Pt bilayer deposition as labelled.
