# Supplementary material for: Modification of dewetting characteristics for the improved morphology and optical properties of platinum nanostructures using a sacrificial indium layer
Source: PLoS One. 2018 Dec 31;13(12):e0209803. doi: 10.1371/journal.pone.0209803 (PMC6312214; doi:10.1371/journal.pone.0209803)
Supplement: S2 Fig — (a) In1.5 nm/Pt4.5 nm, (b) In3 nm/Pt3 nm and (c) In4.5 nm/Pt1.5 nm. (a-1)–(c-1) Corresponding cross-sectional profiles. (DOCX) [file pone.0209803.s002.docx]

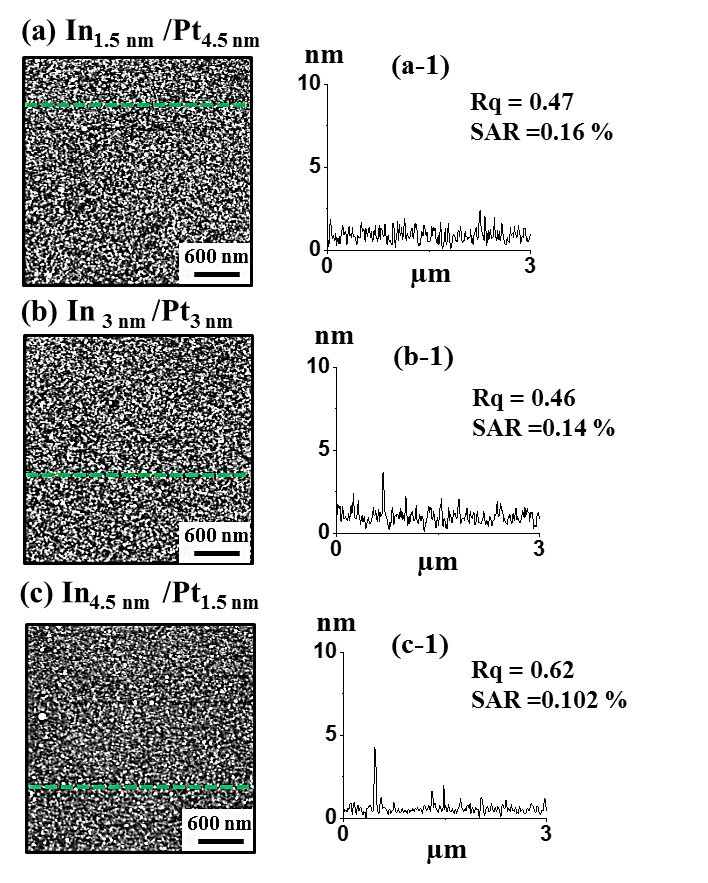


**S2 Fig.** AFM top-views (3 × 3 µm^2^) of samples after the deposition of In-Pt bilayers with various composition: (a) In_1.5 nm_/Pt_4.5 nm_, (b) In_3 nm_/Pt_3 nm_ and (c) In_4.5 nm_/Pt_1.5 nm_. (a-1) – (c-1) Corresponding cross-sectional profiles.
