# Supplementary material for: Modification of dewetting characteristics for the improved morphology and optical properties of platinum nanostructures using a sacrificial indium layer
Source: PLoS One. 2018 Dec 31;13(12):e0209803. doi: 10.1371/journal.pone.0209803 (PMC6312214; doi:10.1371/journal.pone.0209803)
Supplement: S3 Fig — (a)–(d) AFM 3D side-views (1 × 1 μm2). (a-1)–(d-1) Cross-sectional line profiles. (DOCX) [file pone.0209803.s003.docx]

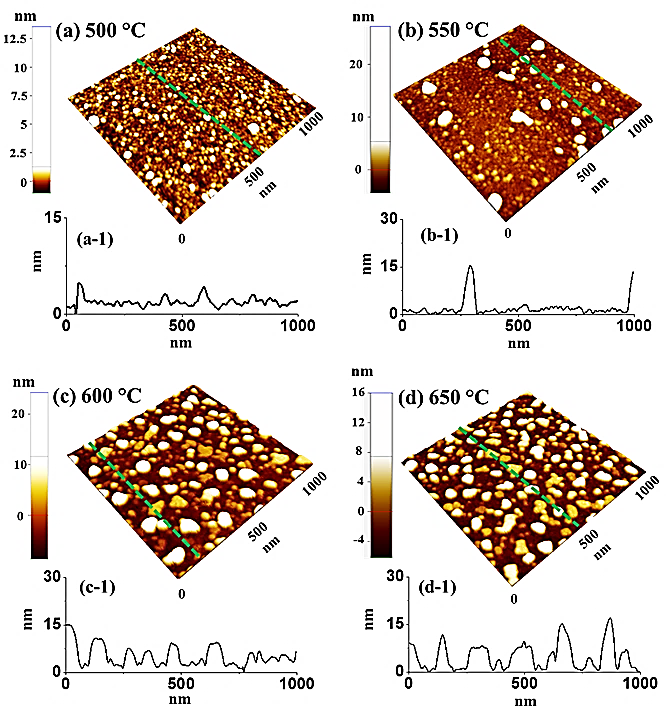


**S3 Fig.** Effect of annealing temperature (500 ºC – 650 ºC) on the morphological evolution of Pt NPs on sapphire (0001) with the In_1.5 nm_/Pt_4.5 nm_ bilayer. (a) – (d) AFM 3D side-views (1 × 1 µm^2^). (a-1) – (d-1) Cross-sectional line profiles.
