# Supplementary material for: Modification of dewetting characteristics for the improved morphology and optical properties of platinum nanostructures using a sacrificial indium layer
Source: PLoS One. 2018 Dec 31;13(12):e0209803. doi: 10.1371/journal.pone.0209803 (PMC6312214; doi:10.1371/journal.pone.0209803)
Supplement: S4 Fig — (a)–(e) AFM 3D side-views (1 × 1 μm2). (a-1)–(e-1) Cross-sectional line profiles. (DOCX) [file pone.0209803.s004.docx]

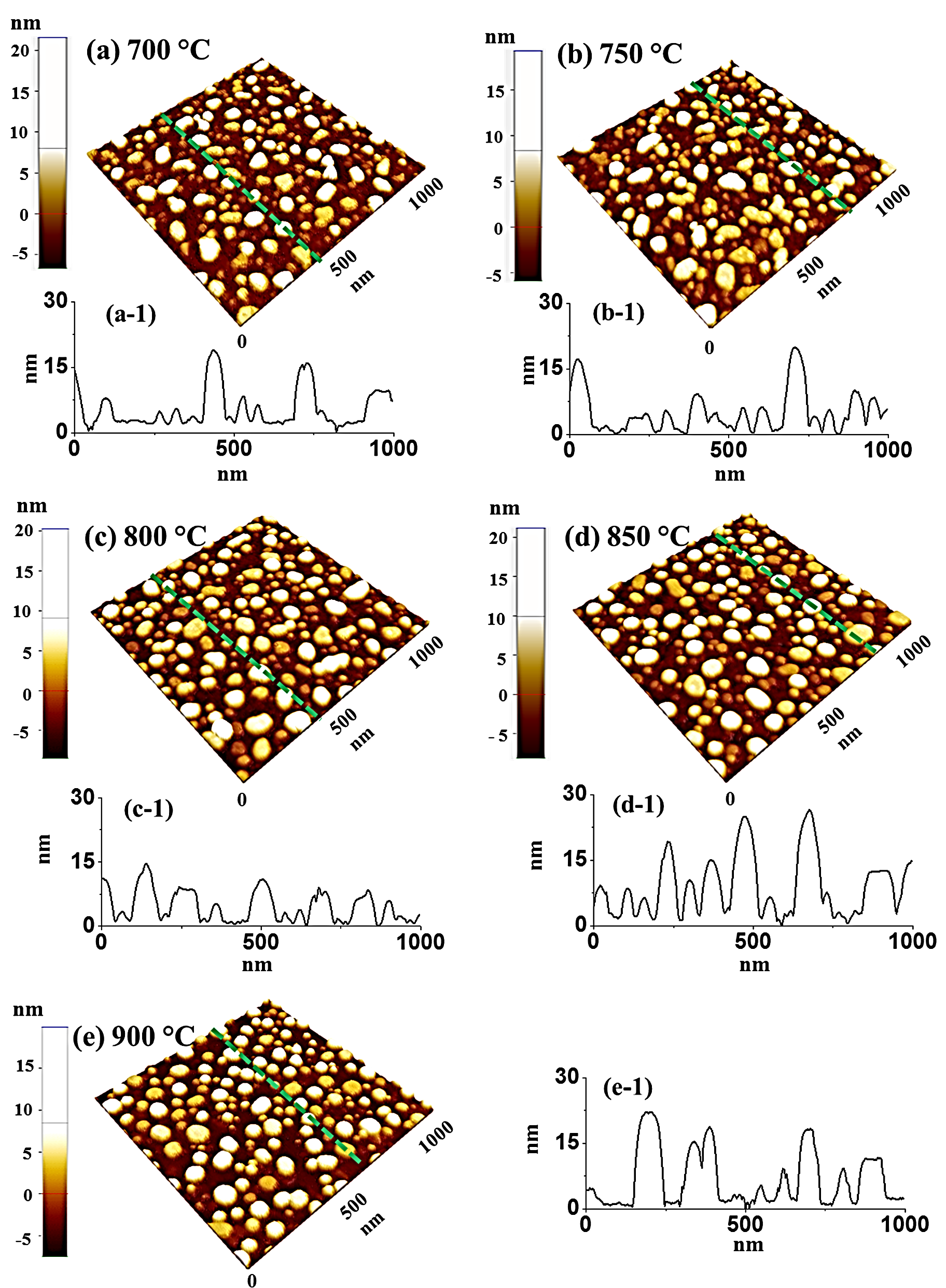


**S4 Fig.** Morphological evolution of Pt NPs on sapphire (0001) with the In_1.5 nm_/Pt_4.5 nm_ bilayer at various annealing temperature (700 ºC – 900 ºC). (a) – (e) AFM 3D side-views (1 × 1 µm^2^). (a-1) – (e-1) Cross-sectional line profiles.
