# Supplementary material for: Modification of dewetting characteristics for the improved morphology and optical properties of platinum nanostructures using a sacrificial indium layer
Source: PLoS One. 2018 Dec 31;13(12):e0209803. doi: 10.1371/journal.pone.0209803 (PMC6312214; doi:10.1371/journal.pone.0209803)
Supplement: S5 Fig — The enlarged views of Pt Mα1 is presented as insets. (DOCX) [file pone.0209803.s005.docx]

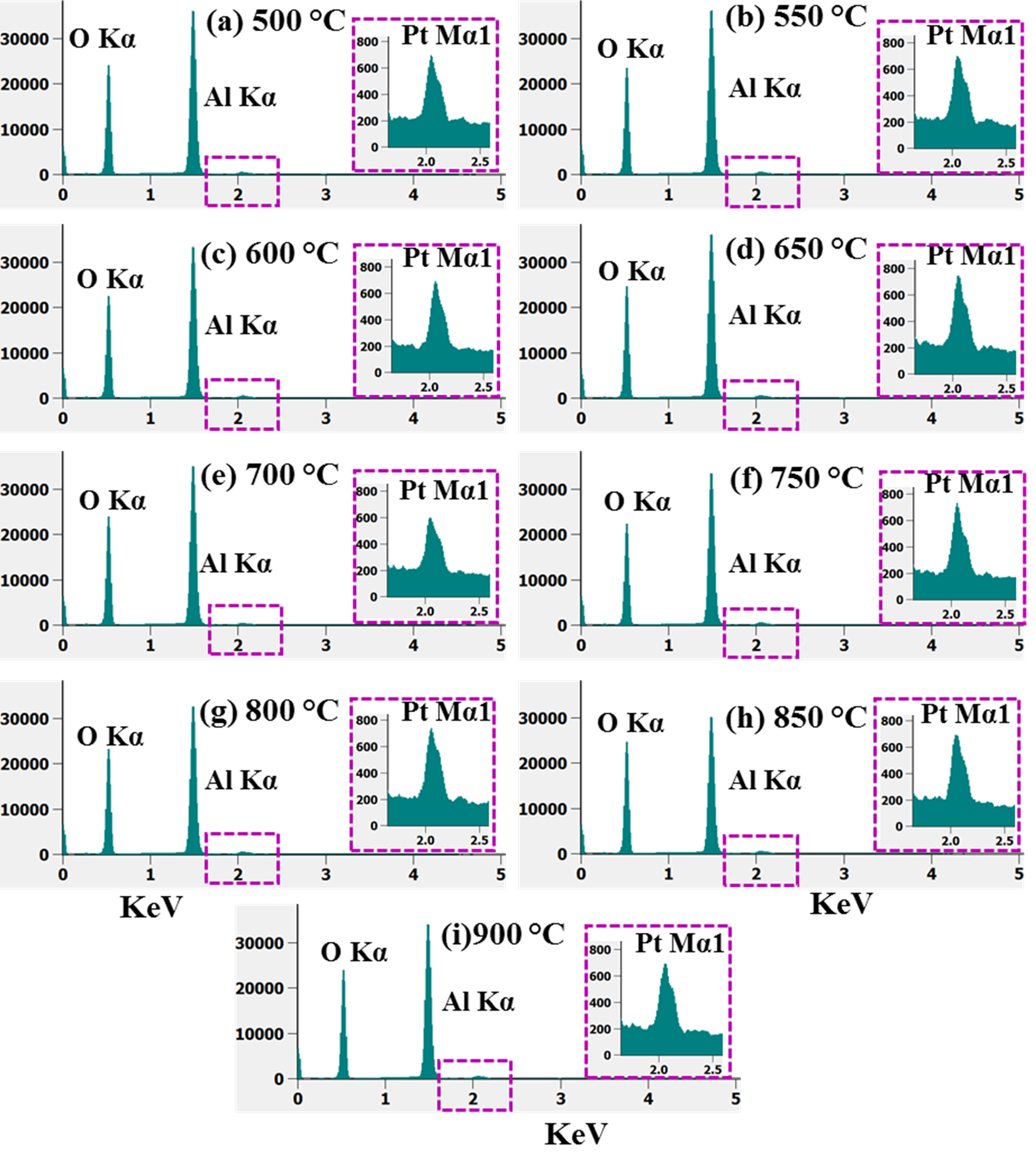


**S5 Fig.** Full range energy-dispersive x-ray spectroscope (EDS) spectra of Pt NPs on sapphire with the In_1.5 nm_/Pt_4.5 nm_ bilayer annealed between 500 and 900 °C for 450 s. The enlarged views of Pt Mα1 is presented as insets.
