# Supplementary material for: Modification of dewetting characteristics for the improved morphology and optical properties of platinum nanostructures using a sacrificial indium layer
Source: PLoS One. 2018 Dec 31;13(12):e0209803. doi: 10.1371/journal.pone.0209803 (PMC6312214; doi:10.1371/journal.pone.0209803)
Supplement: S7 Fig — (a)–(e) AFM D side-views (1 × 1 μm2). (a-1)–(e-1) Cross-sectional line profiles. (DOCX) [file pone.0209803.s007.docx]

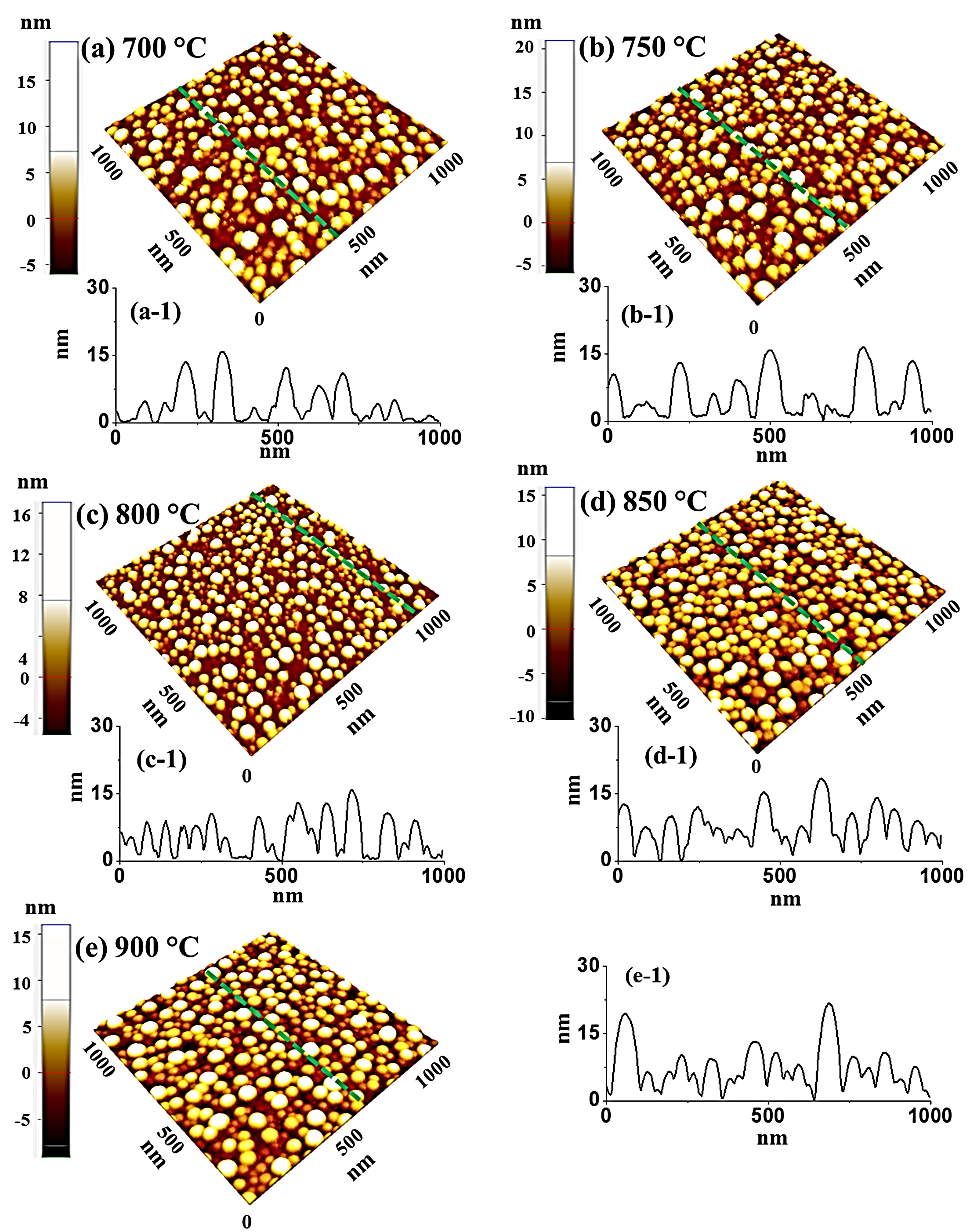


**S7 Fig.** Self-assembled Pt NPs on sapphire (0001) with the fixed bilayer total thickness of 6 nm (In_3 nm_/Pt_3 nm_) by the annealing between 700 and 900 ^o^C for 450 s. (a) – (e) AFM D side-views (1 × 1 µm^2^). (a-1) – (e-1) Cross-sectional line profiles.
