# Supplementary material for: Modification of dewetting characteristics for the improved morphology and optical properties of platinum nanostructures using a sacrificial indium layer
Source: PLoS One. 2018 Dec 31;13(12):e0209803. doi: 10.1371/journal.pone.0209803 (PMC6312214; doi:10.1371/journal.pone.0209803)
Supplement: S8 Fig — Insets show the detail of the Pt Mα1 peak. (DOCX) [file pone.0209803.s008.docx]

+
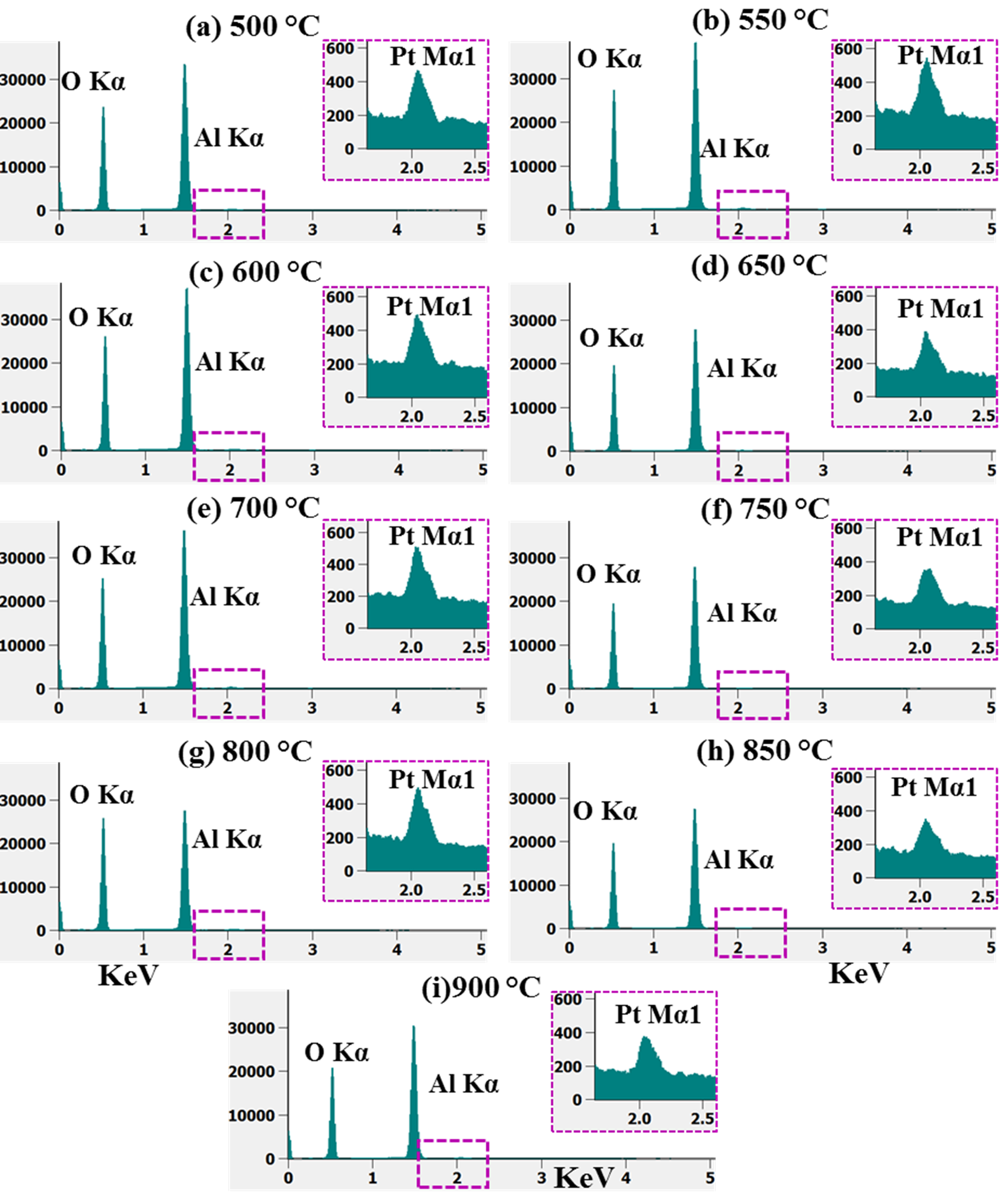


**S8 Fig.** EDS spectra of the Pt NPs on sapphire within the range of 0 - 5 keV annealed at temperatures from 500 to 900 °C for 450 s with the In_3 nm_/Pt_3 nm_ bilayer set. Insets show the detail of the Pt Mα1 peak.
