# Supplementary material for: Modification of dewetting characteristics for the improved morphology and optical properties of platinum nanostructures using a sacrificial indium layer
Source: PLoS One. 2018 Dec 31;13(12):e0209803. doi: 10.1371/journal.pone.0209803 (PMC6312214; doi:10.1371/journal.pone.0209803)
Supplement: S9 Fig — The total bilayer thickness was of 6 nm with the In4.5 nm/Pt1.5 nm bilayer. (a)–(d) AFM side-views (1 × 1 μm2). (a-1)–(d-1) Cross-sectional line profiles. (DOCX) [file pone.0209803.s009.docx]

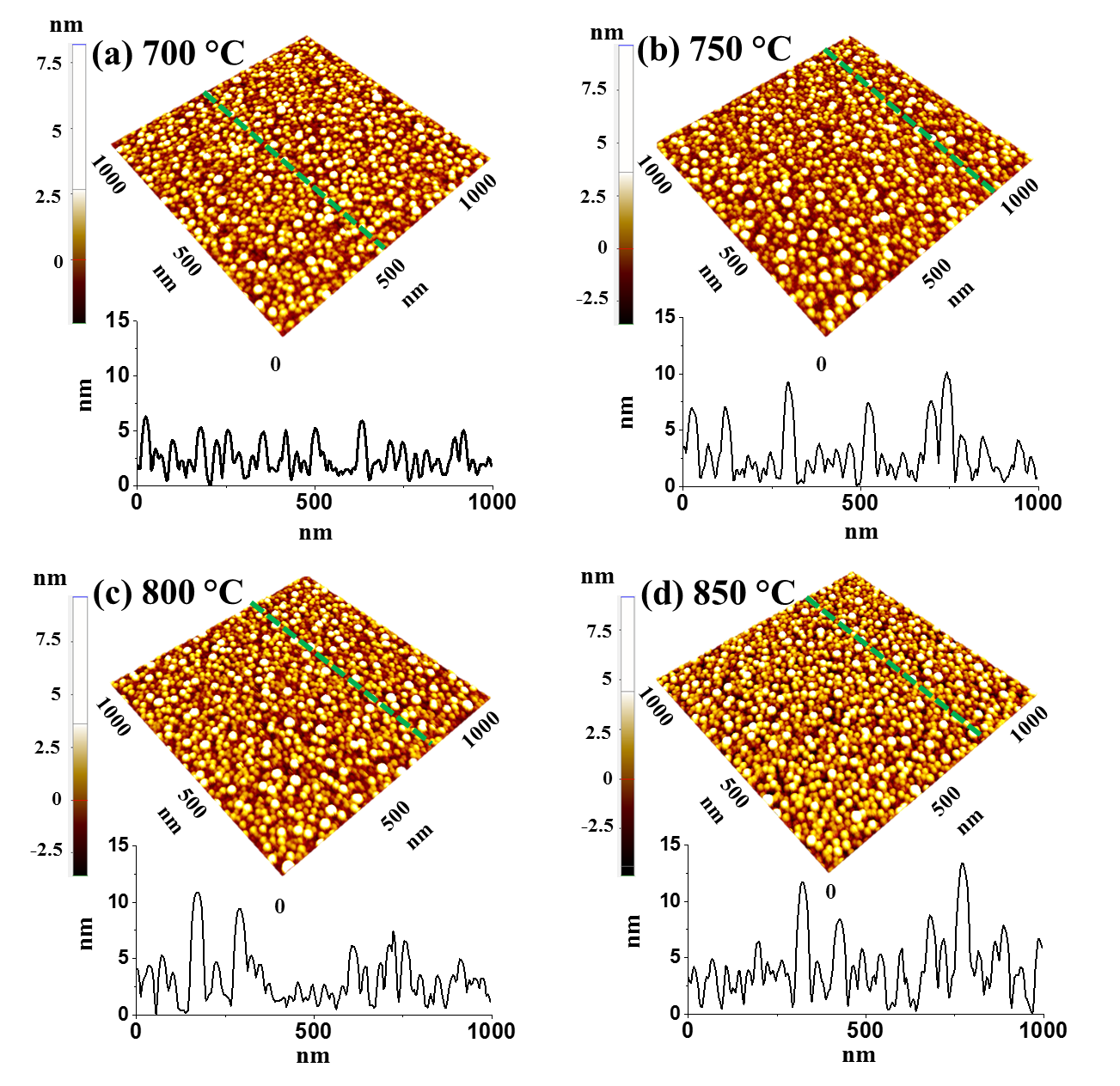


**S9 Fig.** Uniform Pt NPs on sapphire (0001) by the systematic control of annealing temperature from 700 to 850 °C for 450 s. The total bilayer thickness was of 6 nm with the In_4.5 nm_/Pt_1.5 nm_ bilayer. (a) – (d) AFM side-views (1 × 1 µm^2^). (a-1) – (d-1) Cross-sectional line profiles.
