# Supplementary material for: Modification of dewetting characteristics for the improved morphology and optical properties of platinum nanostructures using a sacrificial indium layer
Source: PLoS One. 2018 Dec 31;13(12):e0209803. doi: 10.1371/journal.pone.0209803 (PMC6312214; doi:10.1371/journal.pone.0209803)
Supplement: S10 Fig — The insets show the enlarged Pt Mα1 peaks at each temperature. (DOCX) [file pone.0209803.s010.docx]

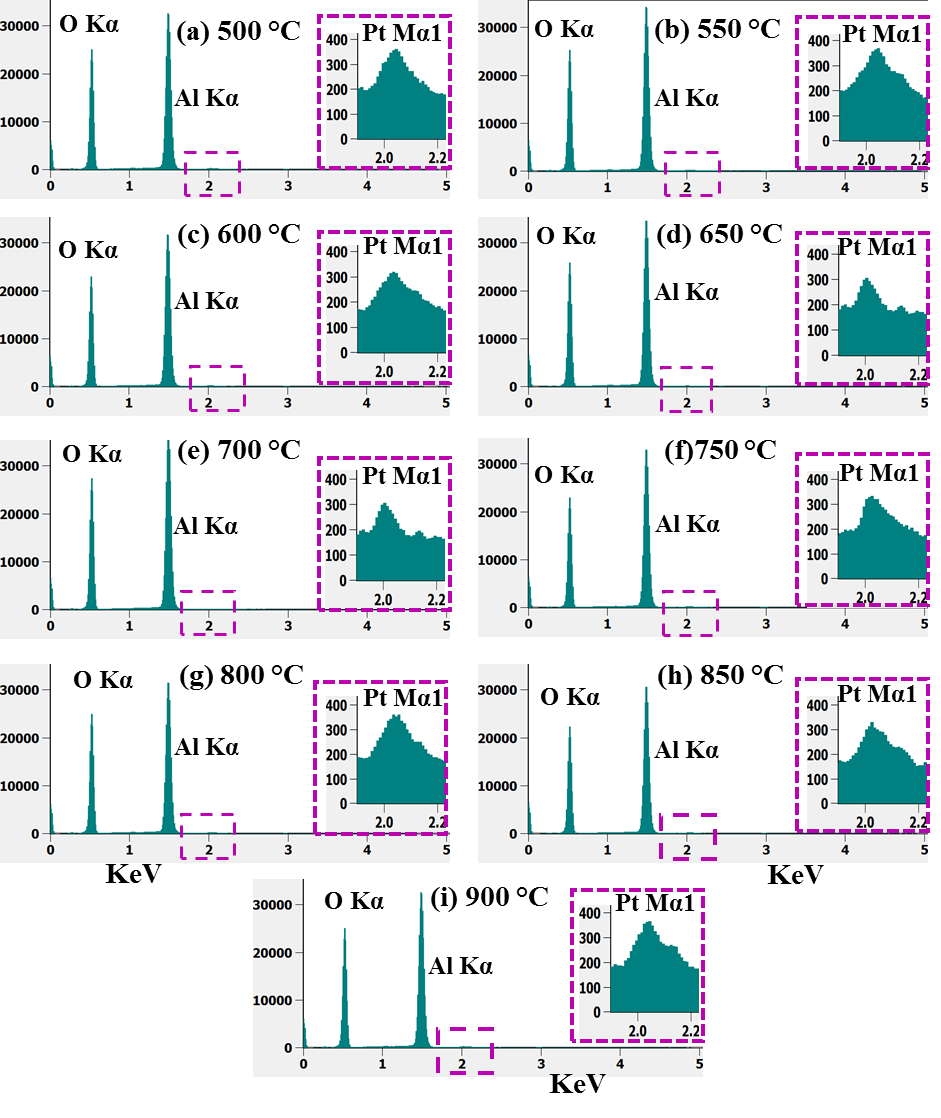


**S10 Fig.** EDS spectra of the Pt NPs on sapphire, fabricated with various annealing temperature as labeled with the In_4.5 nm_/Pt_1.5 nm_ bilayer. The insets show the enlarged Pt Mα1 peaks at each temperature.
