# Supplementary material for: Modification of dewetting characteristics for the improved morphology and optical properties of platinum nanostructures using a sacrificial indium layer
Source: PLoS One. 2018 Dec 31;13(12):e0209803. doi: 10.1371/journal.pone.0209803 (PMC6312214; doi:10.1371/journal.pone.0209803)
Supplement: S1 Table — (DOCX) [file pone.0209803.s011.docx]

**S1 Table.** Summary of root mean squared roughness (Rq), surface area ratio (SAR), average reflectance and transmittance of Pt NPs fabricated between 500 and 900 ºC for 450 s with 6 nm total thickness (In_1.5 nm_/Pt_4.5 nm_).

| **Temperature**  **[°C]** | **Bilayer Thickness (In_1.5 nm_/Pt_4.5 nm_)** | | |  |
| --- | --- | --- | --- | --- |
|  | **Rq [nm]** | **SAR [%]** | **Reflectance [%]** | **Transmittance [%]** |
| **500** | 0.50 | 0.12 | 22.50 | 58.40 |
| **550** | 3.21 | 2.22 | 21.79 | 59.42 |
| **600** | 3.99 | 3.81 | 21.25 | 61.42 |
| **650** | 4.05 | 3.81 | 21.81 | 62.28 |
| **700** | 4.05 | 3.98 | 19.89 | 65.09 |
| **750** | 4.45 | 4.29 | 19.50 | 67.12 |
| **800** | 4.78 | 5.86 | 19.72 | 68.95 |
| **850** | 4.9 | 8.39 | 18.81 | 73.02 |
| **900** | 5.91 | 9.49 | 17.67 | 74.50 |
