# Supplementary material for: Modification of dewetting characteristics for the improved morphology and optical properties of platinum nanostructures using a sacrificial indium layer
Source: PLoS One. 2018 Dec 31;13(12):e0209803. doi: 10.1371/journal.pone.0209803 (PMC6312214; doi:10.1371/journal.pone.0209803)
Supplement: S2 Table — (DOCX) [file pone.0209803.s012.docx]

**S2 Table.** Summary of Rq, SAR, average reflectance and transmittance of Pt NPs fabricated at temperature 500 - 900 ºC for 450 s with the In_3 nm_/Pt_3 nm_ bilayer on sapphire (0001).

| **Temperature**  **[°C]** | **Bilayer Thickness (In_3 nm_/Pt_3 nm_)** | | |  |
| --- | --- | --- | --- | --- |
|  | **Rq [nm]** | **SAR [%]** | **Reflectance [%]** | **Transmittance [%]** |
| **500** | 0.76 | 0.20 | 16.34 | 66.42 |
| **550** | 3.20 | 3.29 | 15.72 | 70.82 |
| **600** | 3.48 | 3.54 | 15.24 | 73.28 |
| **650** | 3.37 | 3.54 | 14.29 | 74.27 |
| **700** | 3.69 | 4.16 | 14.14 | 75.09 |
| **750** | 3.56 | 4.72 | 13.64 | 78.56 |
| **800** | 3.60 | 4.57 | 13.00 | 79.94 |
| **850** | 3.69 | 4.58 | 10.94 | 82.68 |
| **900** | 4.18 | 5.61 | 9.14 | 83.24 |
