# Supplementary material for: Modification of dewetting characteristics for the improved morphology and optical properties of platinum nanostructures using a sacrificial indium layer
Source: PLoS One. 2018 Dec 31;13(12):e0209803. doi: 10.1371/journal.pone.0209803 (PMC6312214; doi:10.1371/journal.pone.0209803)
Supplement: S3 Table — (DOCX) [file pone.0209803.s013.docx]

**S3 Table.** Summary of Rq, SAR, average reflectance and transmittance of Pt nanostructures fabricated between temperature 500 - 850 ºC for 450 s with the In_4.5 nm_/Pt_1.5 nm_ bilayer on sapphire (0001).

| **Temperature**  **[°C]** | **Bilayer Thickness (In_4.5 nm_/Pt_1.5 nm_)** | | |  |  |
| --- | --- | --- | --- | --- | --- |
|  | **Rq [nm]** | **SAR [%]** | **Reflectance [%]** | **Transmittance [%]** |  |
| **500** | 1.33 | 1.37 | 14.55 | 74.01 | |
| **550** | 1.30 | 2.38 | 13.05 | 75.59 | |
| **600** | 1.44 | 2.19 | 12.19 | 77.44 | |
| **650** | 1.45 | 2.94 | 12.47 | 78.01 | |
| **700** | 1.57 | 2.76 | 12.12 | 79.16 | |
| **750** | 1.72 | 3.88 | 12.69 | 79.63 | |
| **800** | 1.84 | 4.56 | 11.25 | 81.41 | |
| **850** | 2.25 | 7.67 | 11.49 | 85.22 | |
